# ACG-Net: an attention context-guided network for fine segmentation of *Fagus* canopies in high-resolution UAV imagery

**DOI:** 10.3389/fpls.2026.1763437

**Published:** 2026-03-23

**Authors:** Yuxin Zhang, Huajuan Gao, Tingting Leng, Xiang Liu, Di Zhang, Zhijie Li, Peng Zhu

**Affiliations:** 1 College of Information Engineering, Sichuan Agricultural University, Ya’an, China; 2 Faculty of Public Administration, Sichuan Agricultural University, Ya’an, China; 3 Key Laboratory of Ecological Forestry Engineering of Sichuan Province, College of Forestry, Sichuan Agricultural University, Chengdu, China

**Keywords:** data augmentation, *Fagus*, forest resource monitoring, remote sensing, semantic segmentation, smart forestry, UAV image segmentation

## Abstract

**Introduction:**

The genus *Fagus*, a key community-forming taxon in northern temperate forests, plays a vital role in maintaining biodiversity and ecosystem functions. However, natural *Fagus* forests are threatened by poor regeneration and community degradation, underscoring the need for precise and efficient monitoring techniques. Existing methods are constrained by the lack of public datasets and the limitations of standard architectures like U-Net in capturing fine-grained features within complex forest scenes.

**Methods:**

To address these challenges, we constructed a high-resolution UAV image segmentation dataset for *Fagus* and proposed the Environment Simulation-based Robust Data Augmentation Framework (ES-REF). By actively simulating realistic disturbances such as fog, local overexposure, and motion blur, ES-REF significantly enhances model generalization under complex conditions. Additionally, we developed ACG-Net, which uses VGG as the encoder backbone and incorporates SPConv, Criss-Cross Attention, and context-guided downsampling to improve multi-scale feature extraction, global context awareness, and spatial detail preservation.

**Results:**

Experimental results demonstrate that ES-REF improves model robustness, increasing the mIoU of U-Net and ACG-Net by 0.71 and 2.18 percentage points, respectively. On the test set, ACG-Net achieved an mIoU of 89.55%, outperforming the U-Net baseline by 4.31 percentage points and surpassing models such as DeepLabv3+.

**Discussion:**

This work establishes a data and methodological foundation for automated *Fagus* community mapping and provides a reliable framework for forest resource monitoring and smart forestry management.

## 1 Introduction

Forests are among the most functionally complex terrestrial ecosystems on Earth, playing a pivotal role in regulating the carbon cycle, conserving biodiversity, and enhancing water retention (Singh and Singh, 2024). *Fagus*, as a key foundation genus in temperate deciduous broadleaf and evergreen–deciduous mixed broadleaf forests across the Northern Hemisphere, exerts a direct influence on ecosystem functioning and stability through the community structure and health of its forests. Under the combined pressures of climate change and human disturbance, natural *Fagus* forests in many regions are exhibiting poor regeneration, community degradation, and localized contractions in distribution range (Xia, 2023). Consequently, fine-scale, dynamic spatial monitoring of *Fagus* forests is essential for developing scientifically sound conservation and restoration strategies. Traditional forest resource surveys have primarily relied on manual field surveys. Although these methods offer high accuracy, they are constrained by limited spatial coverage, low temporal frequency, and high labor and financial costs, making it difficult to support large-scale, high-frequency monitoring (Scott, 1998). In recent years, low-altitude remote sensing technologies, represented by unmanned aerial vehicles (UAVs), have rapidly advanced, enabling the acquisition of centimeter-level, ultra-high-resolution imagery at relatively low cost and providing an unprecedented data foundation for precise species identification and individual-tree-level information extraction (Pajares, 2015).

However, despite the unprecedented data foundation provided by UAV remote sensing, the automatic and accurate segmentation of specific tree species from high-resolution imagery still faces severe challenges at two levels. The first arises from the intrinsic complexity of forest scenes, and the second from the inevitable degradation of image quality during dynamic UAV acquisition. Specifically, the inherent complexity of the scene imposes stringent requirements on the model’s feature representation and discrimination capability. Even within the genus *Fagus*, crown size, morphology, and spectral characteristics exhibit pronounced intraspecific variation due to factors such as tree age and illumination. *Fagus* commonly coexists with other tree species, and substantial interspecific similarity in both morphological and spectral properties further complicates species discrimination. Moreover, complex understory backgrounds (e.g., shrubs, grasses, shadows), together with frequent crown adhesion and occlusion in dense stands, seriously interfere with accurate target identification and precise boundary delineation (Apostol et al., 2020; Ecke et al., 2022; Huang et al., 2023; Chen et al., 2024). In parallel, the dynamic UAV acquisition process in real operations introduces an additional layer of imaging challenges: influenced by gusts and flight-attitude adjustments, aerial images are frequently affected by motion blur or defocus, leading to reduced sharpness and loss of fine details; furthermore, highly variable mountainous weather conditions, such as fog, haze, and strong solar reflections, can reduce image contrast or cause local overexposure, all of which severely compromise model robustness (Sieberth et al., 2014, 2016; Liu et al., 2022; Lee et al., 2024).

The rapid advancement of deep learning technology, particularly the rise of convolutional neural networks (CNNs), has provided powerful tools to address the aforementioned challenges. Since the groundbreaking success of AlexNet (Hinton et al., 2012) in the ImageNet image classification competition in 2012, deep learning models have demonstrated formidable capabilities for automatically learning and extracting hierarchical image features. This breakthrough spurred the development of deeper and more powerful network architectures, such as VGG (Publication, 2012), GoogLeNet (Szegedy et al., 2015), and ResNet (He et al., 2016), which have continually advanced state-of-the-art performance in computer vision tasks.

As deep learning models have advanced, research focus has gradually shifted from image-level classification to more refined pixel-level analysis tasks, namely semantic segmentation. The introduction of Fully Convolutional Networks (FCNs) (Long et al., 2015) marked a milestone in this field, enabling the first end-to-end pixel-level prediction for images of arbitrary size. U-Net (Ronneberger et al., 2015), with its classic encoder–decoder architecture and skip-connection design, effectively integrates high-level semantic information with low-level spatial details, and has become one of the most widely adopted benchmark architectures in medical imaging and remote sensing image analysis.

In recent years, with the rapid advancement of unmanned aerial vehicle (UAV) remote sensing and deep learning technologies, tree species identification based on high-resolution imagery has emerged as a critical research focus for forest resource inventories, forest health assessments, and management operations (Buchelt et al., 2024). While foundational studies established the baseline effectiveness of convolutional neural networks (CNNs) for UAV-based forest analysis, recent research has increasingly focused on overcoming the limitations of standard architectures in complex forest environments through mechanism-level innovations. For example, Marcello et al. systematically evaluated the performance of multiple individual tree segmentation algorithms using UAV LiDAR data across different forest ecosystems, revealing notable performance variations under diverse structural conditions (Marcello et al., 2024). To address the challenge of irregular canopy morphologies, Zeng et al. proposed the FO-Net architecture, which employs adaptive feature extraction mechanisms tailored to variable tree crown structures, leading to notable improvements in segmentation precision for non-standard canopy shapes (Zeng et al., 2025). In parallel, among these mechanism-level innovations, enhanced feature representation has been actively pursued through architectural refinements, such as DMCA-Unet, which integrates multi-scale feature fusion and coordinate attention mechanisms to more effectively capture fine-grained spatial information than conventional U-Net variants (Cai et al., 2025a). More recently, the field has witnessed a paradigm shift toward the adoption of foundation models and novel state-space architectures; notably, the integration of the Segment Anything Model (SAM) with YOLOv10s has demonstrated strong generalization performance in tree detection and instance-level tasks (Shen et al., 2025), while RSVMamba introduces visual state-space modeling into remote sensing analysis, offering a complementary paradigm to CNN-based methods by efficiently modeling long-range dependencies for high-accuracy semantic segmentation (Zhang et al., 2025).

Despite these advancements, critical limitations remain in current research regarding fine-grained canopy segmentation. First, mainstream architectures face challenges in efficient feature representation. Standard convolutions often incur computational redundancy and possess limited multi-scale capabilities, making it difficult to adapt to the pronounced size variations of *Fagus* crowns. Furthermore, architectures like U-Net rely on aggressive downsampling to expand the receptive field, which inevitably results in the over-smoothing of intricate, jagged edges, leading to coarse segmentation where boundaries are blurred and adjacent crowns are falsely merged. Second, standard CNNs are limited in global context modeling, as they utilize local convolutional operations that struggle to capture long-range dependencies. In complex forest backgrounds where *Fagus* is spectrally similar to understory vegetation, local features alone are insufficient for accurate discrimination, often resulting in misclassification within shadowed areas. Finally, there is a notable lack of robustness in existing methods; most models are trained on high-quality datasets and lack specific mechanisms to handle the domain gap caused by environmental interferences—such as fog and motion blur—that are common in mountainous UAV missions (Elizar et al., 2022; Lv et al., 2023). Crucially, beyond these architectural constraints, the performance of deep learning models in this field is severely restricted by the scarcity of large-scale, high-quality annotated datasets. The high cost and difficulty of obtaining pixel-level annotations for complex forest scenes result in a lack of sufficient training data, which directly leads to insufficient segmentation accuracy and limits the models’ ability to generalize across diverse field conditions.

To address the challenges of fine-grained *Fagus* canopy segmentation in high-resolution aerial imagery, this study proposes an integrated framework. The main contributions are summarized as follows:

1. A high-resolution aerial image dataset for fine-scale *Fagus* segmentation is constructed through systematic field investigation and manual annotation.
2. An Environment Simulation-based Robust Data Augmentation Framework (ES-REF) is developed to simulate common environmental degradations and enhance model robustness under real-world acquisition conditions.
3. A novel network, termed ACG-Net, is designed based on the U-Net architecture by incorporating SPConv, Criss-Cross Attention, and Context-Guided Downsampling modules to improve feature representation and spatial detail preservation.

The effectiveness of the proposed framework is validated through extensive experimental evaluation.

## 2 Materials and methods

### 2.1 Study area

**Figure 1:**
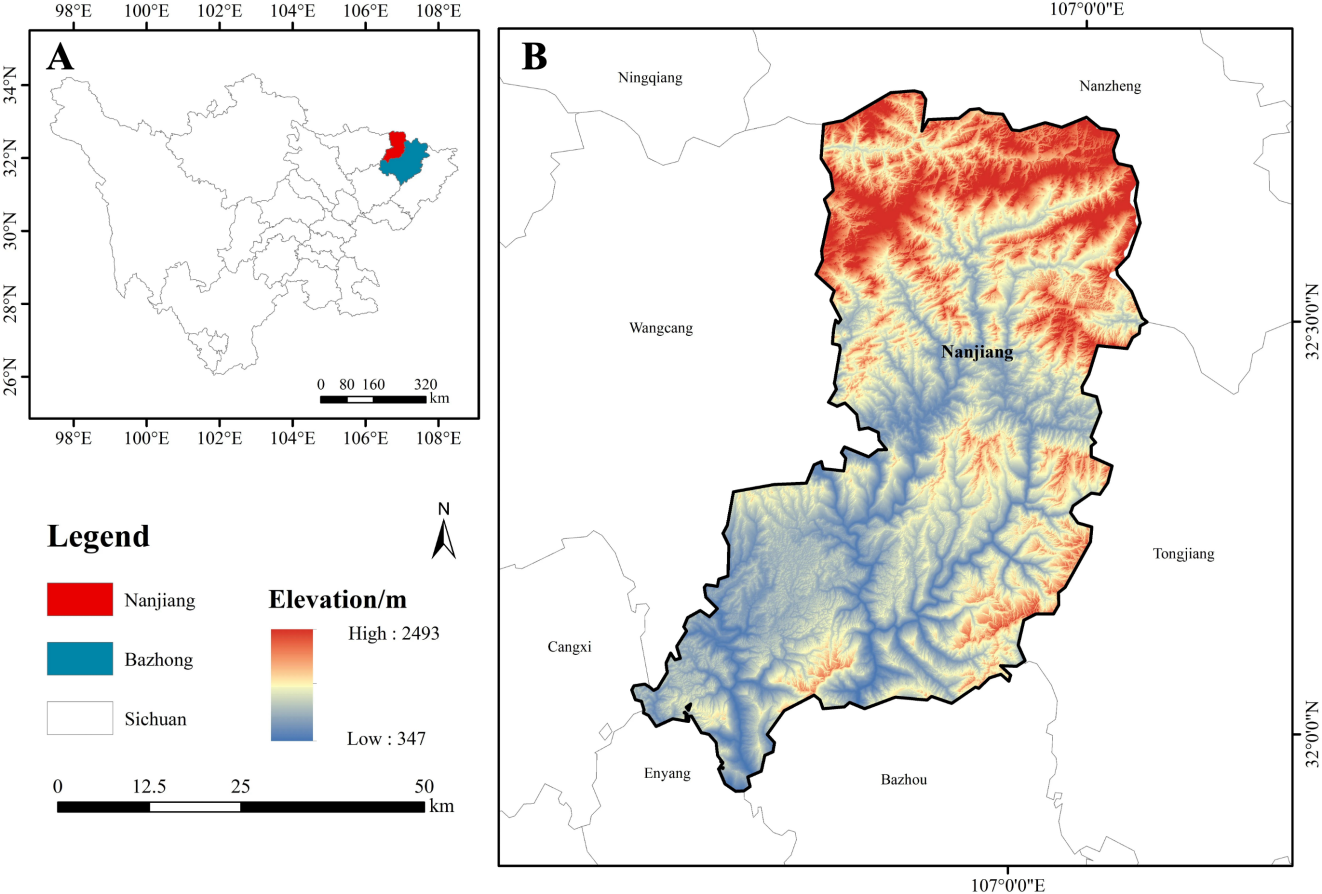
Geographical location and elevation distribution of the study area. **(A)** Location of Nanjiang County within Bazhong City; **(B)** Elevation distribution of the study area, with red areas indicating higher elevations and blue areas indicating lower elevations.

The experimental area of this study is located in the Guangwushan–Micangshan region in Nanjiang County, Bazhong City, Sichuan Province, China (see **Figure 1**), which lies within the Qinling–Daba Mountains. Its geographical coordinates roughly span 106°38′50″–107°05′56″E and 32°31′12″–32°44′29″N, making it a crucial transitional zone between northern and southern China in terms of geography, climate, and ecology. The study area is strongly influenced by both the southeast and southwest monsoons, resulting in a mild and humid subtropical monsoon climate with distinct seasons. The mean annual temperature is approximately 15°C, and annual precipitation exceeds 1,200 mm. These favorable hydrothermal conditions provide an ideal environment for luxuriant forest growth. The primary reason for selecting this area is that the Micangshan region is one of the largest and most species-rich primary forest conservation areas for the genus *Fagus* worldwide. In particular, within the Guangwushan–Daxiaolangou Nature Reserve, several Chinese endemic *Fagus* species are widely distributed, including *Fagus hayatae*, *Fagus engleriana*, *Fagus lucida*, and *Fagus longipetiolata* (Zitao, 2021; Qian, 2024).

Among these, *Fagus hayatae* is the dominant species in this region, primarily distributed at elevations of 1,100–2,200 m and forming extensive pure or mixed forests (Jinxin et al., 2016). The well-preserved and widely distributed *Fagus* populations in this area not only provide a valuable database for ecological studies but also offer sufficient, representative remote-sensing imagery for training and validating the deep learning models used in this study. Additionally, the high density of natural stands and the widespread coexistence of multiple tree species result in typical forest conditions characterized by crown overlap, complex backgrounds, and uneven illumination. These complex real-world conditions present significant challenges to high-precision remote-sensing image segmentation. Nevertheless, they also make the area an ideal testbed for thoroughly assessing the robustness and generalization capability of the proposed Environment Simulation-based Robust Data Augmentation Framework (ES-REF) and ACG-Net under complex conditions.

### 2.2 Data collection and annotation

To ensure the smooth execution of the study, a high-resolution UAV image dataset with sufficient volume and controllable quality was constructed. On November 1–2, 2024, a two-day field acquisition campaign was carried out. During the flights, the weather was clear, with minimal cloud cover or fog and no substantial direct sunlight interference, which ensured the quality of the raw imagery. The data were collected using a DJI Phantom 4 Pro UAV (1-inch CMOS, 20 MP), flying at approximately 100 m above ground level (AGL). Each image achieved a ground sample distance (GSD) of approximately 2 to 3 cm per pixel, providing adequate detail for pixel-level crown segmentation. All raw images were rigorously screened manually to remove those with improper flight altitude, uneven illumination, or insufficient sharpness, resulting in a final base dataset of 1,362 high-quality RGB aerial images.

**Figure 2:**
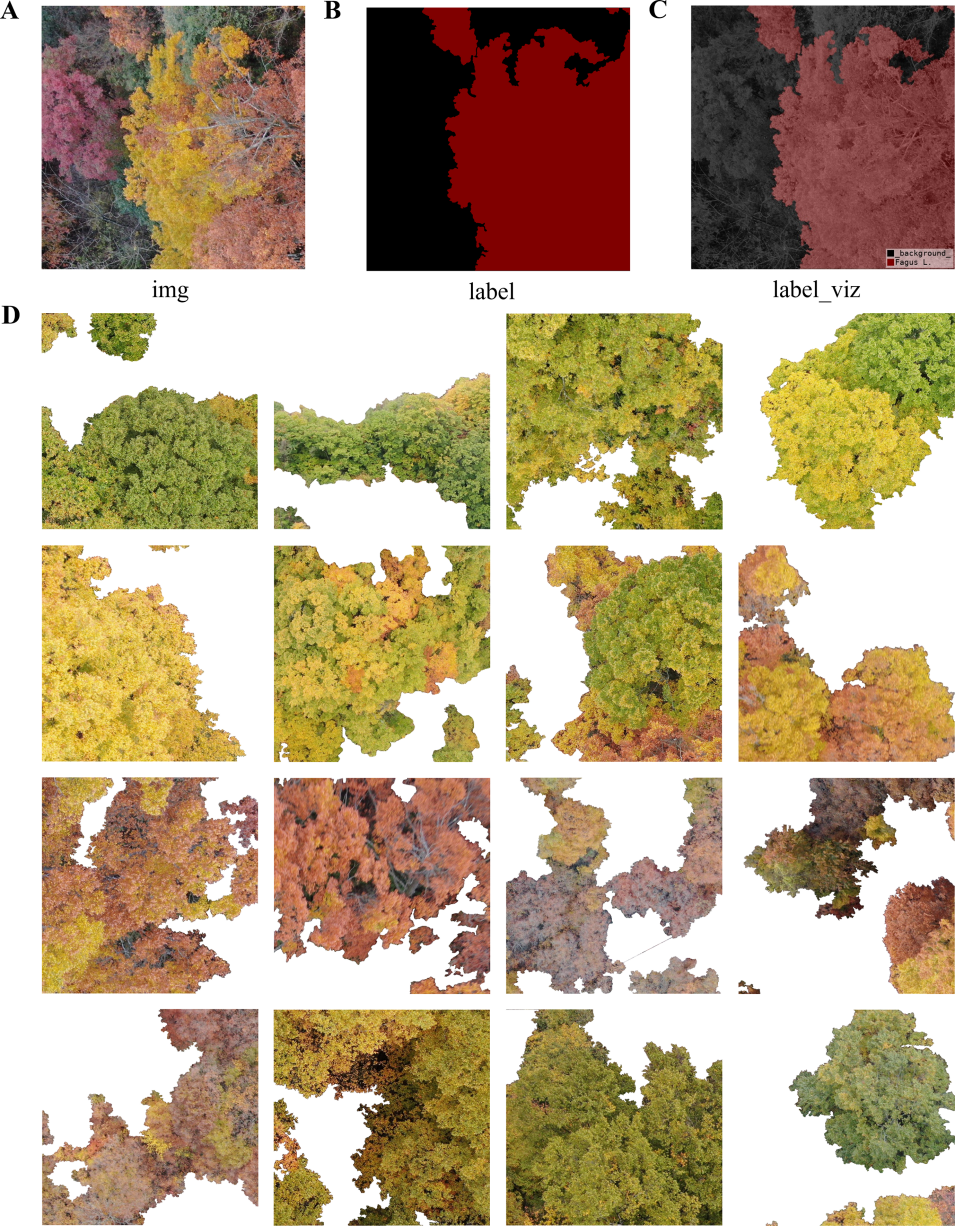
Samples from the fine-grained segmentation dataset of the *Fagus* genus. **(A)** Original aerial image (img); **(B)** Corresponding binary mask (label); **(C)** Mask visualization (label_viz, with the foreground representing the *Fagus* canopy); **(D)** Several representative tree crown image patches randomly selected from the dataset, highlighting the diversity of the *Fagus* genus in terms of morphology, color, crown size, and lighting conditions.

Pixel-level annotations were carried out using Labelme (Russell et al., 2008), defining it as a binary semantic segmentation task (target class: *Fagus*; background class: other objects). The annotations were created by multiple researchers experienced in forestry and remote-sensing image interpretation, who accurately delineated canopy boundaries based on crown morphology, texture, and spectral characteristics. Other objects (e.g., non-target tree species, shrubs, bare soil, rocks, and shadows) were consistently labeled as background. In the label masks, *Fagus* pixels were assigned the value of 1, and background pixels were assigned the value of 0. To guarantee annotation quality, unified annotation guidelines were formulated, and cross-review procedures were applied to maximize accuracy and internal consistency. **Figure 2** shows samples from the fine-grained *Fagus* segmentation dataset that was constructed.

### 2.3 Data augmentation

In the field of deep learning, data augmentation has been widely applied across multiple tasks, such as computer vision and natural language processing, as an effective means to enhance model generalization ability. Traditional data augmentation methods increase the diversity of training samples by applying geometric transformations—including rotation, translation, scaling, and flipping—and color adjustments such as brightness, contrast, and saturation, thereby reducing overfitting to the training set (Shorten and Khoshgoftaar, 2019). For deep learning-based remote sensing image analysis, model performance critically depends on the quality and diversity of the training data. Accordingly, after constructing a high-quality initial dataset, a set of classic, general-purpose augmentation strategies was first introduced in this study. These strategies systematically augment the samples by applying geometric and pixel-level perturbations to the original images, thereby enhancing model robustness under standard conditions and laying the foundation for subsequent, more targeted augmentation and model optimization.

However, although conventional data augmentation techniques can increase the sample size, they primarily consist of simple transformations of idealized data, such as clear images with uniform illumination. As a result, models trained solely on such data often perform poorly when confronted with real, complex, and dynamic field environments. In particular, for forest tree species identification, factors such as crown morphology, illumination variability, and environmental disturbances substantially increase the complexity of remote sensing imagery. This discrepancy is commonly referred to as the domain gap, denoting the difference in model performance between the training domain and the testing domain (Tuia et al., 2016).

When UAVs operate in forested areas, the acquired imagery is affected by uncontrollable factors such as clouds, sudden illumination changes, flight jitter, and occlusions, which characterize the real data domain and constitute major sources of the domain gap. To bridge this gap, the issue is addressed at its source by shifting the paradigm from simple data augmentation to explicit environment simulation and reproduction. Unlike traditional data augmentation methods, the proposed framework goes beyond merely expanding ideal data by actively generating and simulating complex samples and corner cases that are difficult to obtain at scale under real-world conditions. This enables the model to learn and adapt to these real-world challenges during training (Tremblay et al., 2018).

Against this background, simple transformations such as conventional geometric and color perturbations are no longer sufficient to capture the diversity and complexity inherent in forest tree species identification tasks. For accurate recognition of forest tree species, more targeted data augmentation strategies are required—strategies that explicitly account for the complexity of forest environments and emphasize the model’s adaptability to disturbances commonly encountered under real-world operating conditions. Therefore, environment simulation techniques are introduced to generate training samples that more faithfully reflect actual operating conditions by simulating clouds and fog, localized overexposure, dynamic motion blur, and random occlusions, thereby narrowing the domain gap and improving model performance in practical applications. In this way, the diversity of the dataset is enhanced, and the model is enabled to maintain high robustness and accuracy under complex real-world environments (Zang et al., 2019).

#### 2.3.1 Traditional data augmentation methods

**Figure 3:**
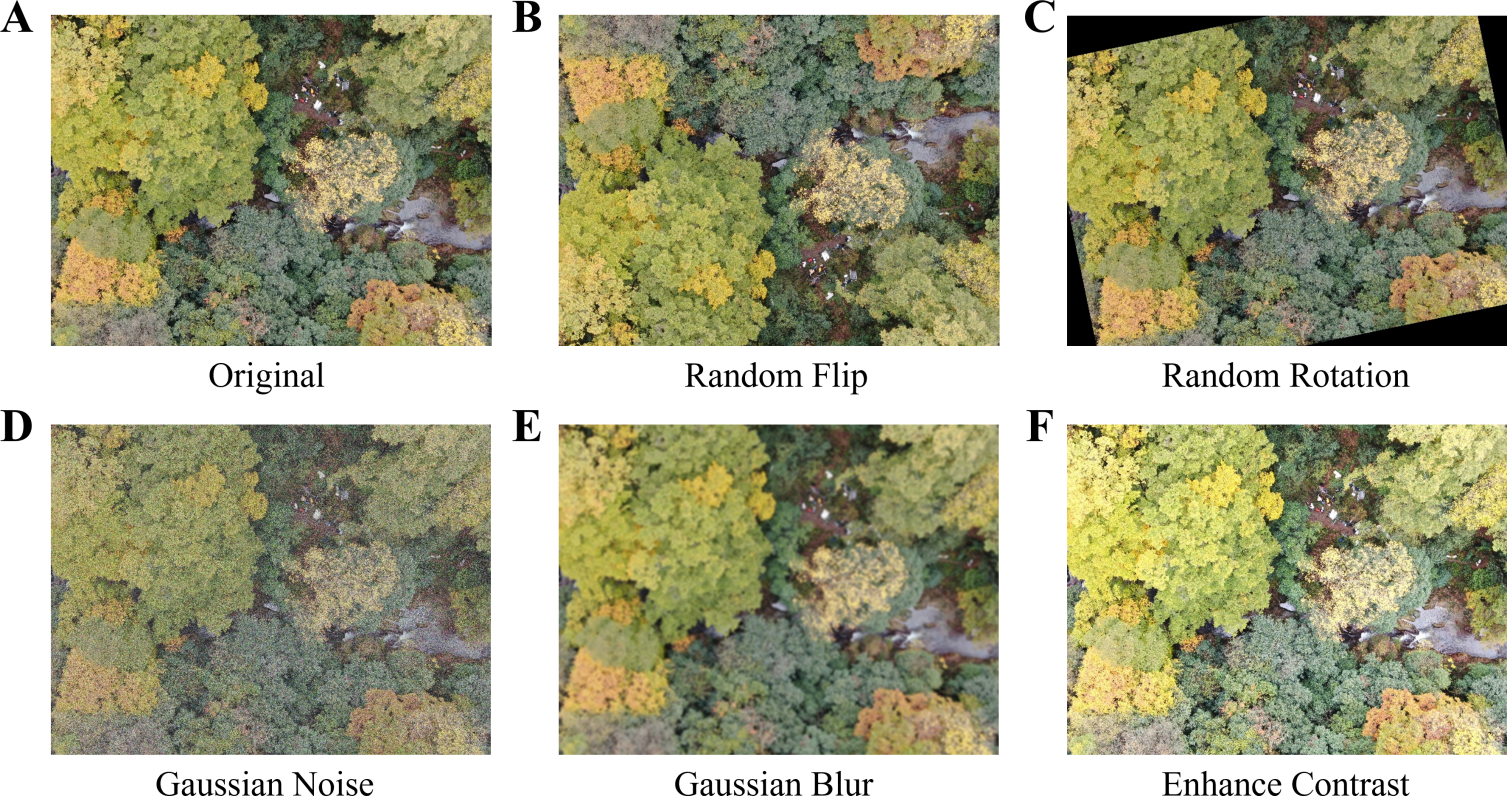
Traditional data augmentation methods. **(A)** Original image; **(B)** Random Flip; **(C)** Random Rotation; **(D)** Gaussian Noise; **(E)** Gaussian Blur; **(F)** Enhance Contrast.

To enhance the robustness of semantic segmentation models against common geometric transformations and pixel-level perturbations, a set of classic, easy-to-implement data augmentation operations was first applied during the data preprocessing stage (see **Figure 3**). These operations included geometric transformations such as flipping and rotation, as well as pixel-level perturbations such as noise addition, blurring, and contrast adjustment. The core objective was to increase the observational diversity of the training samples, enabling the model to learn invariance or equivariance to common imaging transformations and thereby reduce overfitting to the training set. All augmentation operations were applied synchronously to both the images and their corresponding masks. During geometric transformations, the masks were subjected to the same spatial operations, whereas during pixel-level perturbations, the masks were kept unchanged to ensure label–pixel consistency.

In terms of geometric transformations, the primary objective is to simulate variations in the spatial position and orientation of objects, thereby enhancing the model’s invariance to object pose and spatial location. For top-down UAV remote sensing imagery, tree canopy orientation is essentially arbitrary. Therefore, two geometric transformation operations were primarily adopted in this study: Random Flipping and Random Rotation.

At the pixel level, perturbations are primarily introduced to simulate variable real-world illumination conditions and sensor noise, thereby enhancing model robustness to changes in lighting and image quality. In this study, three typical pixel-level perturbation operations were adopted: Gaussian Noise, Gaussian Blur, and Contrast Adjustment.

#### 2.3.2 Environment simulation-based robust data augmentation framework

**Figure 4:**
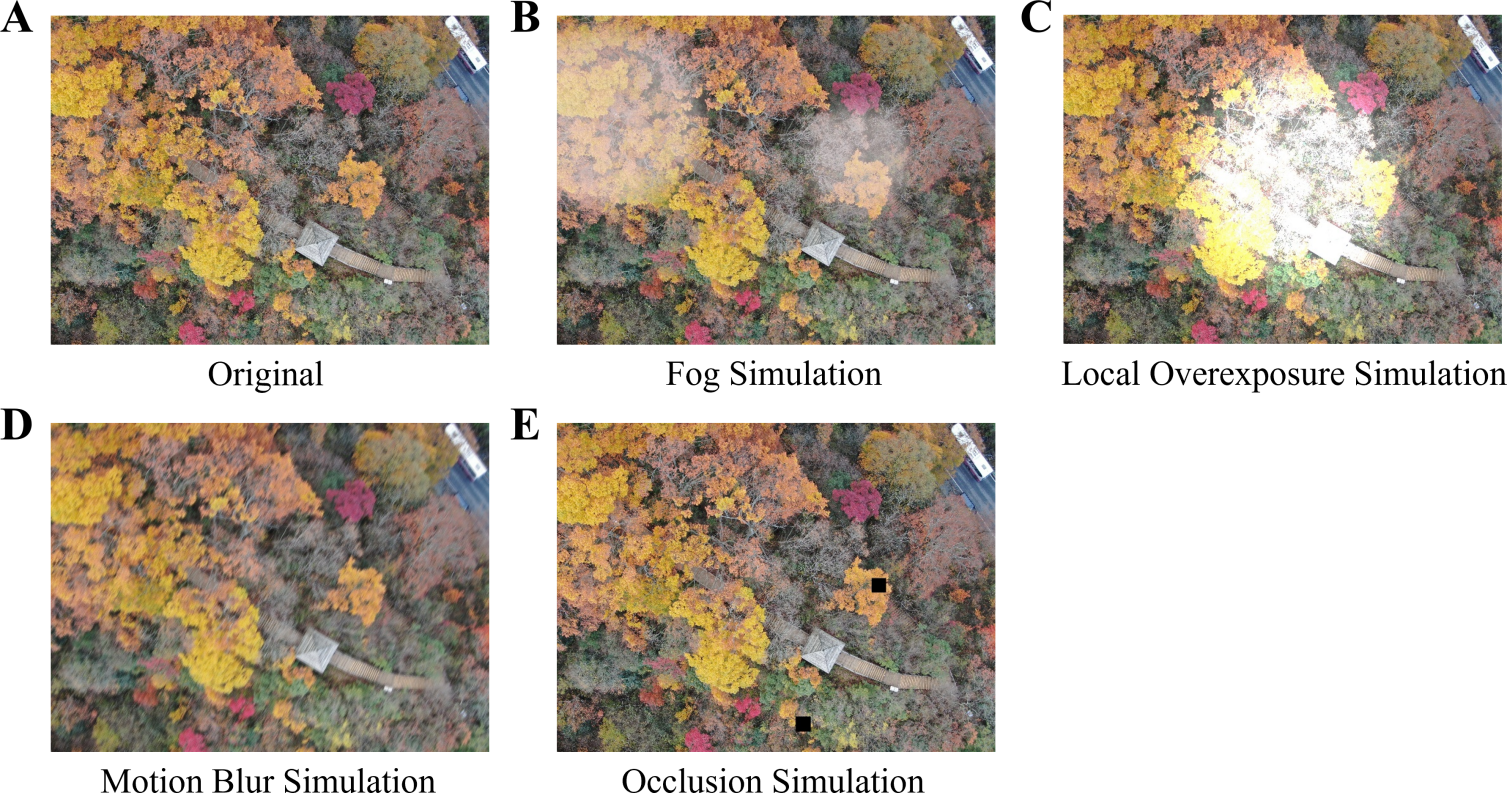
Environment simulation-based robust data augmentation. **(A)** Original image; **(B)** Fog Simulation; **(C)** Local Overexposure Simulation; **(D)** Motion Blur Simulation; **(E)** Occlusion Simulation.

An Environment Simulation-based Robust Data Augmentation Framework (ES-REF) has been designed and proposed in this study to specifically address the problem of insufficient training data in complex scenarios. The core concept of this framework involves compensating for the scarcity of real-world samples by proactively generating challenging samples using algorithmic methods that are likely to induce model misclassification. This forces the model to learn more robust deep features that are insensitive to imaging degradation and environmental interference, thereby enhancing its feature invariance across multiple dimensions, including illumination, clarity, and completeness. To achieve this goal, ES-REF utilizes a set of highly targeted simulation modules. Each module replicates a typical physical or operational challenge and expands the dataset diversity through parameter randomization, thereby covering the critical differences between the training domain and the real-world domain (see **Figure 4**). To ensure reproducibility, the specific parameter ranges and implementation details for these simulation modules are provided in **Table 1**.

**Table 1:** Detailed parameter settings for the ES-REF framework.

Module	Parameter	Notation	Value/Range
Fog Simulation	Opacity Range	*α*	[0.4,0.8]
	Fog Color (RGB)	$C_{fog}$	[220,245]
	Spot Radius	$R_{fog}$	Adaptive(0.3×min(H, W))
	Gaussian Kernel Size	*k*	$Adaptive(0.7\times R_{fog})$
	Number of Spots	*N*	[1,3]
Local Overexposure Simulation	Highlight Radius	*R*	Adaptive(0.25 × min(H, W))
	Exposure Intensity Coefficient	*α*	[0.5,0.9]
	Maximum Brightness Increment	*β*	[150,255]
	Gaussian Kernel Size	*k*	Adaptive(1.5×R)
Motion Blur Simulation	Kernel Length	*L*	[5,31] pixels
	Angle	*θ*	$\left[0^{\circ },180^{\circ }\right)$
	Mixing Coefficient	*γ*	[0.35,0.8]
Occlusion Simulation	Patch Size	*S*	50×50 pixels
	Fill Value	*k*	0 (Black)

(1)Fog Simulation

In simulating haze or scattered light scenes, ES-REF approximates contrast attenuation and high-frequency detail loss caused by atmospheric scattering by constructing a local transparency map and linearly blending it with a preset fog color. The specific approach involves randomly sampling *N* (where N∈[1,3]) circular regions on the image plane, applying Gaussian filtering to smooth the generated opacity mask (creating a continuous transparency map $M_{fog}$), then weighting the original image with the fog color $C_{fog}$ to produce the enhanced result. This method aims to reduce the model’s reliance on fine textures, encouraging it to focus on more robust canopy contours and structural features. The transformation formula is given in Equation 1:

(1) $$\begin{array}{c} I^{\prime }(x,y,c)=clip((1-M_{fog})I(x,y,c)+C_{fog}M_{fog},0,255) \end{array}$$

Where I(x,y,c) denotes the pixel value of the original image at spatial position (x,y) and channel c; $I^{\prime }(x,y,c)$ denotes the corresponding pixel value of the augmented image after haze or scattered-light simulation. $M_{fog}$ is the transparency coefficient map obtained through Gaussian smoothing. Its peak opacity values (denoted as α) are randomly selected within the range [0.4,0.8]. the fog color $C_{fog}$ is randomly generated within a high-brightness white range [220,245] to simulate different illumination conditions. Finally, clip(·) constrains pixel values to the range [0,255].

(2) Local Overexposure Simulation

To address brightness distortion caused by local overexposure or strong light reflections, ES-REF designed a light occlusion simulation module based on brightness superposition. This module randomly generates a local highlight mask, applies Gaussian smoothing to create a gradual brightness field $M_{field}$, and performs additive enhancement, thereby simulating the loss of detail resulting from overexposure. The specific transformation formula is given in Equation 2:

(2) $$\begin{array}{c} I^{\prime }(x,y,c)=clip(I(x,y,c)+\beta M_{field}(\alpha ),0,255) \end{array}$$

where β represents the Maximum Brightness Increment, which is randomly selected within the range [150,255] to control the peak magnitude of the added light. $M_{field}(\alpha )$ denotes the smoothed brightness field, where the initial opacity of the mask is determined by the α, randomly sampled from [0.5,0.9] to ensure natural saturation levels. The smoothing kernel size is dynamically adapted based on the R.

(3) Motion Blur Simulation

Drones are often affected by factors such as gusts of wind or sudden attitude adjustments during flight, which can cause rapid displacement of the camera at the moment of exposure, leading to directional motion blur. Unlike traditional Gaussian blur, this type of blur has a distinct direction and is closely related to the displacement path of the camera during exposure. To simulate this effect, ES-REF developed a dynamic motion blur simulation module. This module generates linear convolution kernels with random directions and lengths and applies them to filter the image, accurately simulating the physical motion blur effect caused by the camera’s linear path during exposure. In the simulation process, the size of the blur kernel $L\in [L_{min},L_{max}]$ and the rotation angle $\theta \in [0^{\circ },180^{\circ })$ are randomly generated. Then, a weighted blending method is used to combine the blurred image with the original image, thereby simulating the motion blur effect. This method realistically reproduces the directional motion blur caused by linear displacement or camera jitter during exposure, testing the model’s boundary retention and segmentation robustness under airborne dynamic disturbances and when fine details, such as branches and leaves, are stretched. The specific transformation formulas are given in Equations 3 and 4:

(3) $$\begin{array}{c} B(x,y,c)=conv(I(x,y,c),psf(x,y)) \end{array}$$

(4) $$\begin{array}{c} I^{\prime }(x,y,c)=clip((1-\gamma )I(x,y,c)+\gamma \cdot B(x,y,c),0,255) \end{array}$$

where B(x,y,c) is the motion-blurred image, obtained by convolving with the rotated linear kernel; psf(x,y) is the point spread function, representing the generated linear convolution kernel used to describe the motion blur path during the camera’s movement. The size and direction of the blur kernel are determined by the randomly generated parameters L and θ. $L_{min}$ and $L_{max}$ represent the minimum and maximum lengths (in pixels) of the blur kernel, while γ is the intensity mixing coefficient, controlling the blending ratio between the blurred image and the original image.

(4) Occlusion Simulation

In practical UAV imaging, foreground branches, birds, or other flying objects may temporarily occlude the lens or target, resulting in missing canopy information. To simulate this scenario, a random occlusion module based on the Cutout strategy was introduced in ES-REF. A rectangular region R is randomly selected on the image, and the pixels within this region are set to a constant value *k* (DeVries and Taylor, 2017). This method is explicitly designed to simulate foreground occlusion, encouraging the model to develop stronger inference capabilities by inferring the overall canopy structure from incomplete visible regions, thereby improving the completeness and robustness of the segmentation results. The transformation formula is given in Equation 5:

(5) $$\begin{array}{c} I^{\prime }(x,y,c)=\{\begin{array}{ll} k, & if(x,y)\in R\\ I(x,y,c), & otherwise \end{array} \end{array}$$

where *R* is the randomly selected occlusion region with a fixed size S×S, and k is the fill value for the occlusion.

#### 2.3.3 Dataset splitting and processing

The dataset was split into training and validation sets at a final 8:2 ratio after data augmentation, with augmentation applied exclusively to the training set to prevent data leakage between the two subsets. The final dataset comprised 4510 samples, including 3605 training images and 905 validation images, with no separate test set constructed. The original images had a spatial resolution of 4000 × 3000 pixels. To improve computational efficiency and ensure compatibility with the U-Net architecture, all images were uniformly resized to 512 × 512 pixels, thereby standardizing the input dimensions and achieving a balance between computational cost and model performance.

### 2.4 Model design

#### 2.4.1 ACG-Net

**Figure 5:**
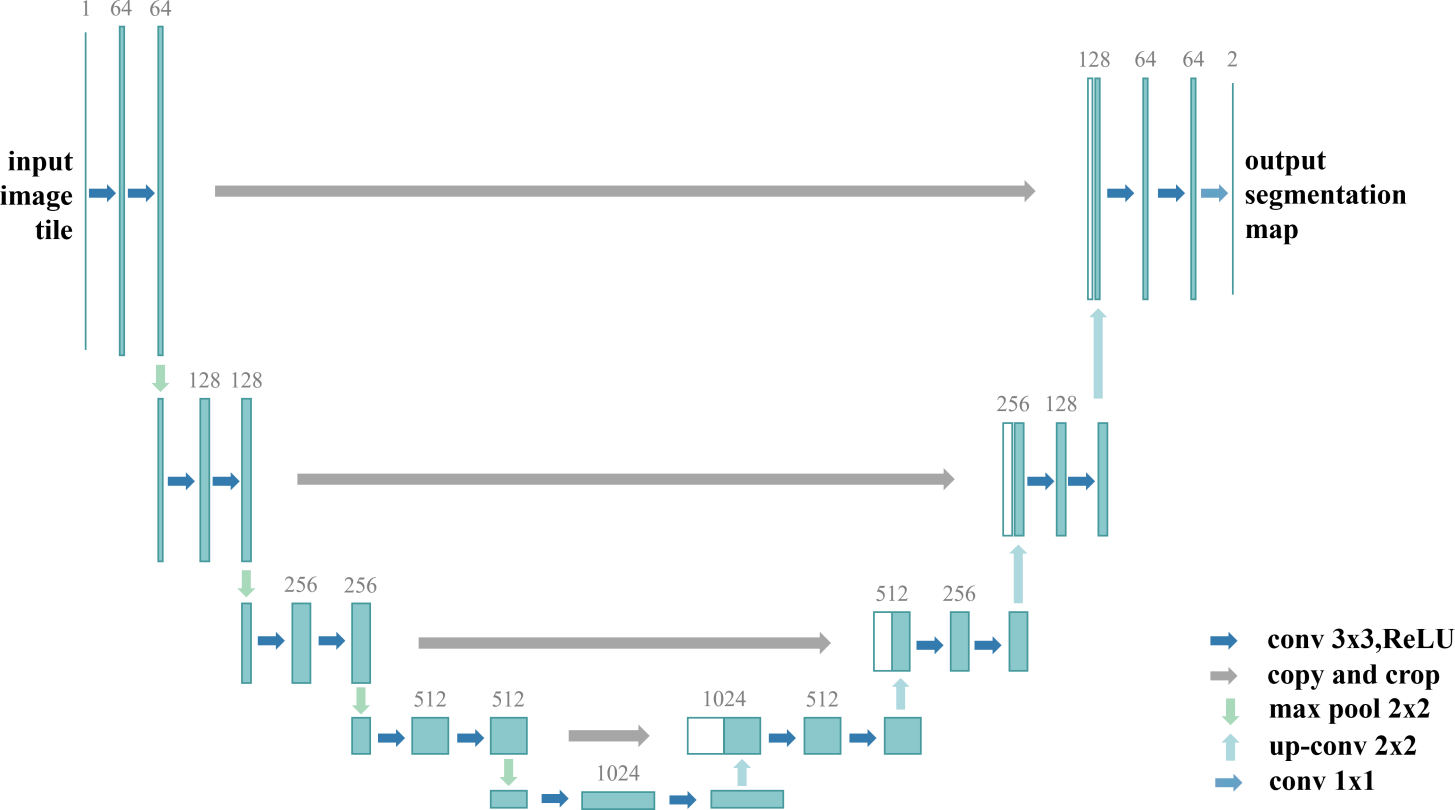
U-Net structure diagram.

U-Net is a classical and widely used semantic segmentation architecture, originally developed as an extension of fully convolutional networks (FCNs). It is named “U-Net” because its overall architecture resembles the letter “U”. Originally designed for biomedical image segmentation, U-Net maintains high accuracy even with limited training samples and has therefore been widely applied in domains such as remote sensing image analysis. As shown in **Figure 5**, U-Net adopts a symmetric encoder–decoder architecture that integrates shallow spatial features with deep semantic features through skip connections, thereby effectively mitigating information loss during downsampling.

**Figure 6:**
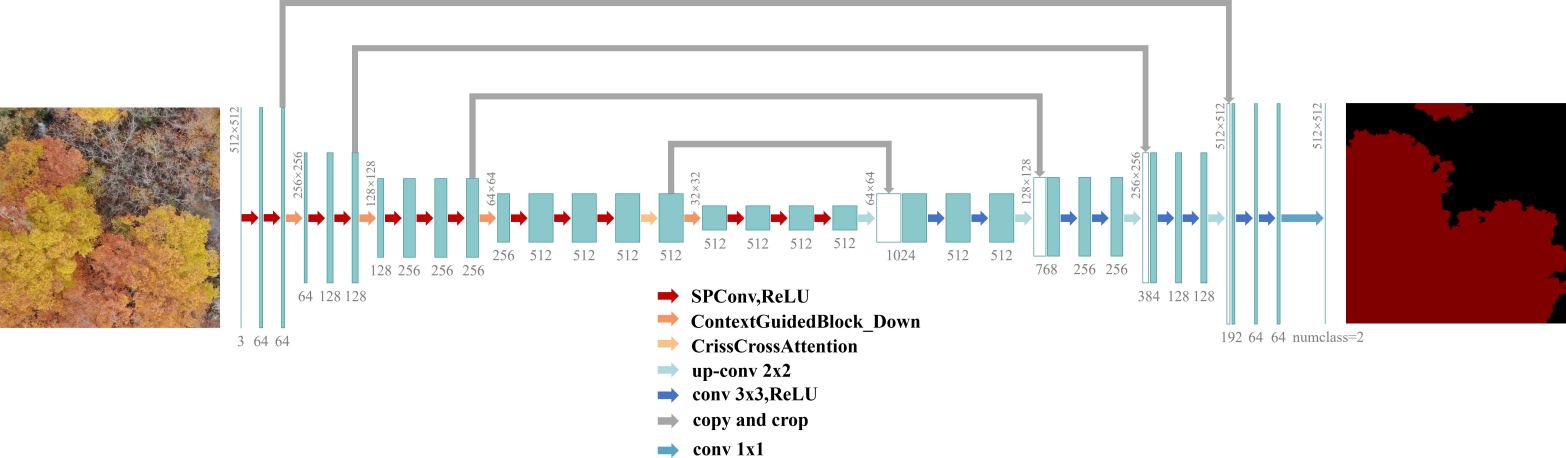
ACG-Net structure diagram.

In this study, an enhanced network, ACG-Net, is proposed, which is specifically optimized for high-resolution remote sensing imagery and built upon the U-Net architecture. As illustrated in **Figure 6**, the core design of ACG-Net centers on the structural optimization of three key components of the U-Net encoder: convolutional feature extraction, contextual information modeling, and spatial detail preservation.

First, the SPConv module is employed to replace the standard convolutional layers in the encoder. This module reduces feature redundancy in high-resolution images through channel splitting and feature fusion strategies, thereby improving the network’s feature extraction efficiency and multi-scale representation capability. Next, the Criss-Cross Attention module is integrated into the high-level semantic feature maps of the encoder. By establishing cross-attention in both the horizontal and vertical directions, it efficiently models global dependencies, thereby significantly enhancing the network’s ability to recognize and segment targets in complex forest backgrounds. Finally, the Context-Guided Block Down module is used to replace the max-pooling layers, enabling context-guided adaptive downsampling and better preserving critical spatial details, such as canopy edges.

#### 2.4.2 SPConv module

In deep convolutional neural networks, traditional convolution, also known as vanilla convolution, uses kernels that operate jointly across all input channels. However, extensive research shows that as network depth increases, significant pattern redundancy often arises among feature maps across different channels; that is, multiple feature maps may learn and encode highly similar texture or structural information. For the *Fagus* canopy segmentation task in this study, the high image resolution and complex backgrounds exacerbate this redundancy, leading to two prominent issues: first, substantial computational and parameter overhead, as high-resolution inputs sharply increase the convolutional layers’ floating-point operations (FLOPs) and parameter count; second, inefficient feature representation, as repeatedly convolving similar features wastes computation and fails to enhance the model’s ability to distinguish subtle differences. Therefore, reducing redundancy in convolution operations while preserving representational capacity becomes crucial to optimizing the network architecture.

**Figure 7:**
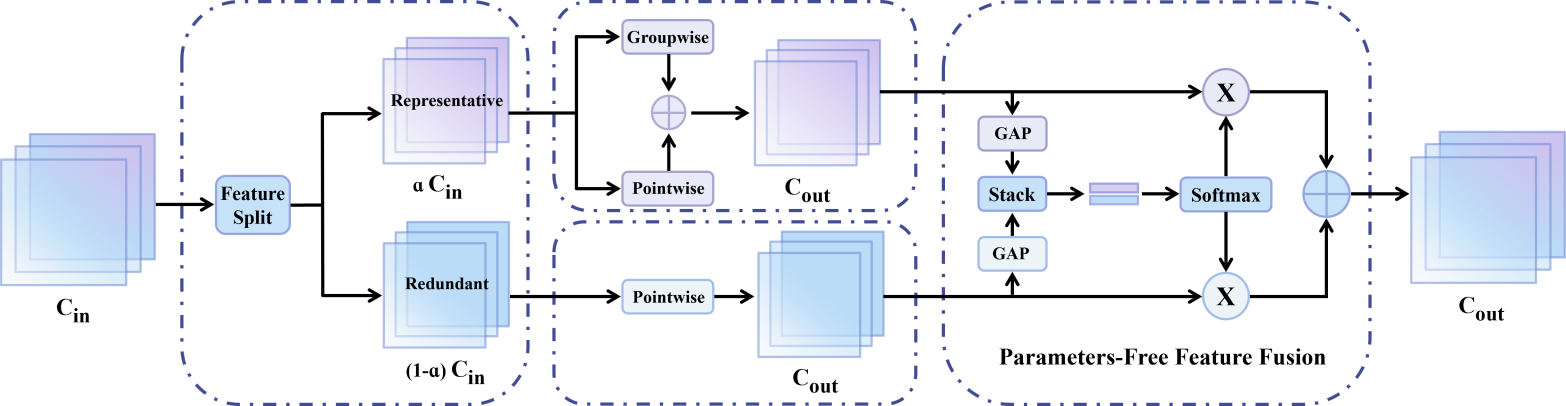
SPConv module.

To address the aforementioned issues, this study incorporates the SPConv (Split-based Convolution) module proposed by Zhang et al. (2020) into the encoder section of U-Net, as shown in **Figure 7**. Unlike conventional methods that focus on eliminating unimportant filters, SPConv focuses on solving the often-overlooked problem of feature map pattern redundancy. Zhang et al. pointed out that while many feature maps within a layer exhibit similar patterns, they may still contain distinct detailed information. Removing these maps directly would lead to information loss. To address this, SPConv proposes an innovative channel-splitting convolution strategy: instead of simply removing redundant features, it divides the input channels into several groups and performs convolutions separately. This enables the model to tolerate similar patterns while significantly reducing the overall computational load, thereby achieving a balance between efficiency and performance.

(1) Vanilla Convolution

Let $X\in R^{L\times h\times w}$ and $Y\in R^{M\times h\times w}$ represent the input and convolved output tensors, with L input channels and M output channels, respectively. Typically, a k×k convolution kernel $W\in R^{L\times k\times k\times M}$ is used to convolve the L input channels into M output channels for feature extraction, resulting in Y=WX+b. For simplicity, we omit the bias term and consider only a single spatial location, in other words, the k×k region covered by the kernel. The expression for the full h×w feature map can be obtained in the same way. Therefore, traditional convolution can be expressed as Equation 6:

(6) $$\begin{array}{c} \left[\begin{array}{c} y_{1}\\ y_{2}\\ \vdots \\ y_{M} \end{array}]=[\begin{array}{cccc} W_{11} & W_{12} & \cdots & W_{1,L}\\ W_{21} & W_{22} & \cdots & W_{2,L}\\ \vdots & \vdots & \ddots & \vdots \\ W_{M,1} & W_{M,2} & \cdots & W_{M,L} \end{array}][\begin{array}{c} x_{1}\\ x_{2}\\ \vdots \\ x_{L} \end{array}\right] \end{array}$$

where $x_{1}$, $x_{2}$,…, $x_{L}$ denote the input feature map elements, $y_{1}$, $y_{2}$,…, $y_{M}$ denote the output feature map elements, and $W_{ij}$, i, j∈[1;L,M] is one of the parameters of the *M* convolution kernels.

(2) The Representative and the Redundant

Assuming the feature map has *L* channels, the SPConv module partitions them according to the ratio α into two parts: representative channels ( αL) and redundant channels ( (1−α)L). The representative branch preserves the main structural and semantic information and first applies a k×k convolution (typically 3×3) to extract the primary features. The redundant branch contains detail features that are related to the primary information but differ only slightly; it uses a lightweight 1×1 convolution to capture the necessary supplementary details at minimal cost, without significantly increasing computation. Therefore, the basic formulation of SPConv is expressed in Equation 7:

(7) $$\begin{array}{c} \left[\begin{array}{c} y_{1}\\ y_{2}\\ \vdots \\ y_{M} \end{array}]=[\begin{array}{ccc} W_{11} & \cdots & W_{1,\alpha L}\\ \vdots & \ddots & \vdots \\ W_{M,1} & \cdots & W_{M,\alpha L} \end{array}][\begin{array}{c} x_{1}\\ \vdots \\ x_{\alpha L} \end{array}\right] \end{array}+[\begin{array}{ccc} w_{1,\alpha L+1} & \cdots & w_{1,L}\\ \vdots & \ddots & \vdots \\ w_{M,\alpha L+1} & \cdots & w_{M,L} \end{array}][\begin{array}{c} x_{\alpha L+1}\\ \vdots \\ x_{L} \end{array}]$$

where $W_{ij}$, j∈[1,αL], denotes the 3×3 convolution kernel parameters applied to the αL representative channels; and $w_{ij}$, j∈[αL+1,L], denotes the 1×1 (pointwise) convolution kernel parameters applied to the remaining (1−α)L redundant channels.

(3) Further Reduction for the Representative

After partitioning all input channels into two parts, to further reduce redundancy in the representative channels, the SPConv module introduces Groupwise Convolution (GWC), dividing the representative channels into several subgroups, each of which performs independent convolutions to reduce computational complexity. However, group convolution breaks connections between channels, which may result in the loss of useful information. To address this issue, the SPConv module adds a 1×1 convolution (Pointwise Convolution, PWC) on all representative channels to restore the information interaction between channels. Unlike conventional methods, the SPConv module performs GWC and PWC operations in parallel on the same group of representative channels and merges these two features through direct addition, ultimately producing the output (with a group size set to 2). Therefore, the representative part in Equation 7 can be expressed as Equation 8:

(8) $$\left[\begin{array}{ccc} W_{11}^{p} & 0 & 0\\ 0 & \ddots & 0\\ 0 & 0 & W_{GG}^{p} \end{array}][\begin{array}{c} z_{1}\\ \vdots \\ z_{G} \end{array}]\begin{array}{c} +[\begin{array}{ccc} w_{11} & \cdots & w_{1,\alpha L}\\ \vdots & \ddots & \vdots \\ w_{M,1} & \cdots & w_{M,\alpha L} \end{array}] \end{array}[\begin{array}{c} x_{1}\\ \vdots \\ x_{\alpha L} \end{array}\right]$$

where G denotes the number of groups. The αL representative channels are partitioned into G groups, each containing $\frac{\alpha L}{G}$ channels. $z_{1}$, $z_{2}$,…, $z_{G}$ are the input features for each group after the partition, and $W_{ll}^{p}$ is the parameter of the convolution kernel for the l-th group.

(4) Parameter-Free Feature Fusion Module

After the preceding steps, two types of features are obtained, originating from the representative branch and the redundant branch, respectively. Because these features arise from different input channel groups, a fusion mechanism is required to regulate the information flow. Global Average Pooling (GAP) is adopted in the SPConv module to produce channel-wise statistics $S_{1}$, $S_{3}\in R^{C}$ for global information embedding. By reducing the spatial dimensions of the output feature U, the c-th element of S can be written as Equation 9:

(9) $$\begin{array}{c} S_{kc}=F_{ɡap}\left(U_{kc}\right)=\frac{1}{H\times W}\sum_{i=1}^{H}\sum_{j=1}^{W}U_{kc}\left(i,j\right),k\in [1,3] \end{array}$$

where $S_{kc}$ denotes the global-pooling statistic of the c-th channel in the k-th branch, $F_{ɡap}$ is the Global Average Pooling operator, $U_{kc}$ is the feature map of the c-th channel in the k-th branch, H and W denote the height and width of the feature map, and C is the total number of channels.

Then, the two resulting $S_{3}$, and $S_{1}$ vectors are stacked, followed by a soft attention operation across channels to generate the feature importance vectors $\beta \in {\text{R}}^{c}$ and $\gamma \in {\text{R}}^{c}$, the c-th element of which, are given in Equation 10:

(10) $$\begin{array}{c} \beta_{c}=\frac{e^{S_{3c}}}{e^{S_{3c}}+e^{S_{1c}}},\gamma_{c}=1-\beta_{c} \end{array}$$

where $\beta_{c}$ and $\gamma_{c}$ denote the attention weights for the c-th channel in the redundant and representative channel branches, respectively; $S_{3c}$ and $S_{1c}$ are the global pooling statistics for the c-th channel in the redundant and representative channel branches, respectively.

The final output Y is given in Equation 11, obtained by fusing features $U_{3}$ and $U_{1}$, from the representative and redundant parts, directed by the feature importance vector β and γ in a channel-wise manner:

(11) $$\begin{array}{c} Y=\beta U_{3}+\gamma U_{1} \end{array}$$

where Y denotes the fused output feature map; β and γ are channel-wise importance vectors generated by the attention mechanism, corresponding to the fusion weights for the redundant and representative branches, respectively.

Through branch processing, group convolutions, lightweight pointwise convolutions, and attention-based fusion, SPConv significantly reduces parameter count and computational cost while enhancing the network’s capacity to model complex features. In high-resolution UAV imagery, the SPConv module more effectively models boundary details of small-scale targets (e.g., tree canopies), thereby avoiding redundant computation and mitigating accuracy degradation in complex backgrounds. Finally, regarding implementation details, SPConv is designed to directly replace standard convolutional layers in the encoder. Given an input feature map $X\in {\text{R}}^{C_{in}\times H\times W}$, the module produces an output feature map $Y\in {\text{R}}^{C_{out}\times H\times W}$. Notably, the spatial resolution ( H×W) remains strictly unchanged during this process, ensuring that the module focuses solely on channel-wise feature refinement without altering geometric dimensions.

#### 2.4.3 Criss-cross attention module

In addition to the aforementioned feature redundancy issue, the inherent locality of traditional convolution operations presents another core limitation. Due to the dependence of the receptive field on stacking convolutional layers, standard CNNs struggle to efficiently capture long-range dependencies between distant pixels in an image. This limitation is particularly prominent in the aerial *Fagus* identification task of this study: when the texture features of the target canopy closely resemble those of surrounding vegetation, relying solely on local information for classification can easily lead to confusion between the target and the background, resulting in blurred or incomplete segmentation boundaries.

**Figure 8:**
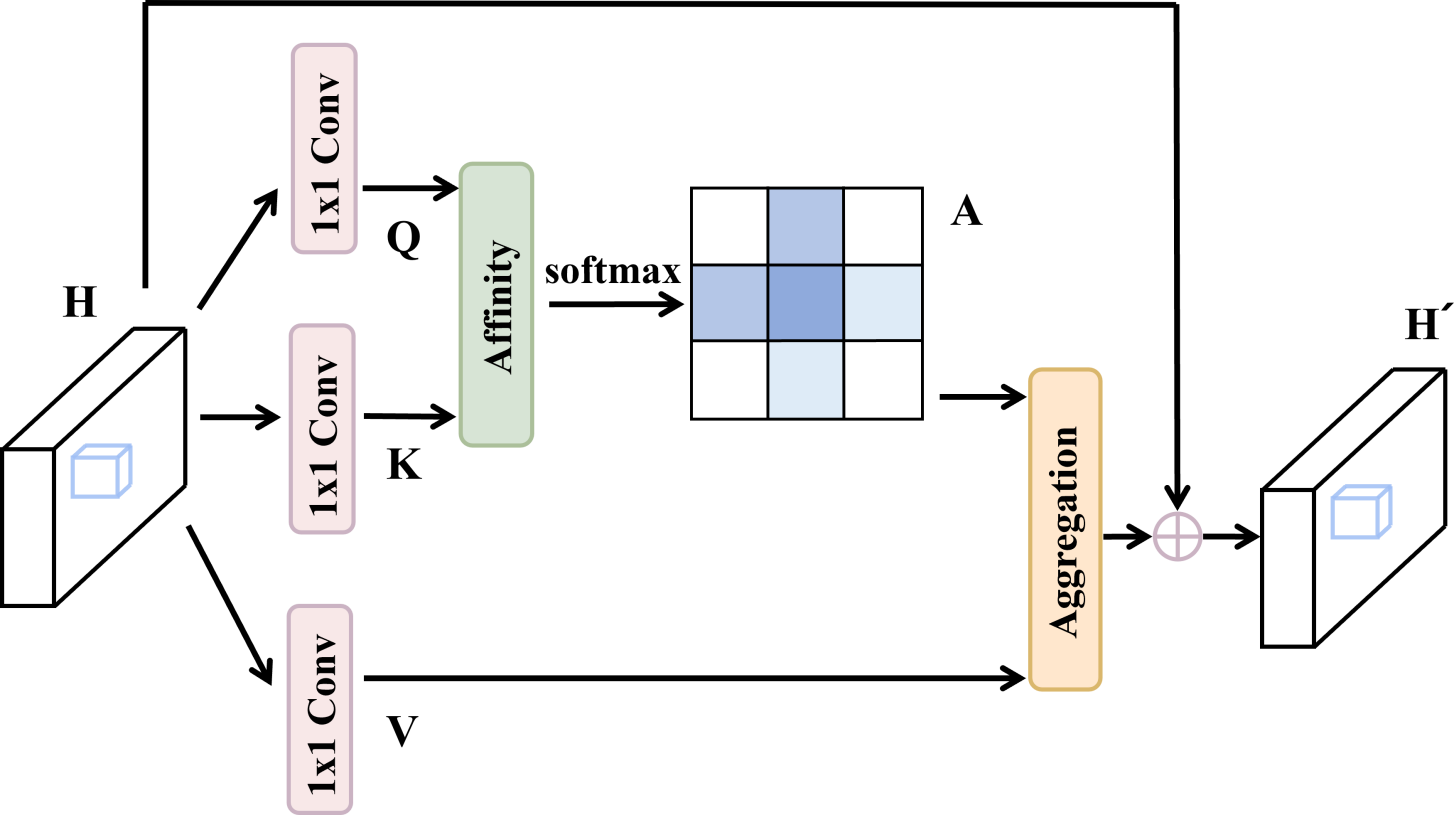
Criss-Cross attention module.

To overcome this limitation, the optimal solution is to enable the network to model global contextual dependencies. Non-local attention can achieve this goal, but it requires calculating a dense attention matrix of size H×W×H×W across the entire feature map, causing both computational complexity and memory usage to grow quadratically with the size of the feature map. For high-resolution aerial imagery, this computational cost becomes prohibitive. Therefore, this study introduces the Criss-Cross Attention module as a more efficient alternative (Huang et al., 2018), illustrated in **Figure 8**. This module employs a sparse attention mechanism to efficiently aggregate global contextual information at a computational cost significantly lower than that of non-local attention.

The Criss-Cross Attention module aggregates contextual information in both horizontal and vertical directions, thereby enhancing pixel-level representation. As shown in the figure below, given a local feature map $\text{H}\in R^{C\times W\times H}$, this module first applies two 1×1 convolutions on H, generating two feature maps, Q and K, where $Q,K\in R^{C^{\prime }\times W\times H}$. Here, $C^{\prime }$ represents the number of channels, which is smaller than C for dimensionality reduction.

After obtaining Q and K, the Criss-Cross Attention module further generates an attention map $A\in R^{(H+W-1)\times H\times W}$ through an Affinity operation. For each position u in the feature map Q, a vector $Q_{u}\in R^{C^{\prime }}$ can be obtained. At the same time, feature vectors located in the same row or column as position u in K are extracted to form the set ${\text{Ω}}_{u}\in R^{(H+W-1)\times C^{\prime }}$. ${\text{Ω}}_{i,u}\in R^{C^{\prime }}$ is the i-th element of ${\text{Ω}}_{u}$. The definition of the Affinity operation is as follows, given in Equation 12:

(12) $$\begin{array}{c} d_{i,u}=Q_{u}{{\text{Ω}}_{i,u}}^{⊺} \end{array}$$

where $d_{i,u}\in D$ represents the correlation measure between the feature $Q_{u}$ and ${\text{Ω}}_{i,u}$, $i=[1,\ldots ,|{\text{Ω}}_{u}|]$, and $D\in R^{(H+W-1)\times H\times W}$.

Then, the Criss-Cross Attention module applies a softmax layer on D along the channel dimension to compute the attention map A. Next, a 1×1 convolution layer is applied to H to generate $\text{V}\in {\text{R}}^{C\times W\times H}$ for feature adaptation. At each position u in the feature map V, a vector $V_{u}\in {\text{R}}^{C}$ can be obtained, along with a set ${\text{Φ}}_{u}\in {\text{R}}^{(H+W-1)\times C}$. The set ${\text{Φ}}_{u}$ consists of feature vectors from V that are in the same row or column as position u. Finally, an Aggregation operation is used to collect contextual information, as given in Equation 13:

(13) $$\begin{array}{c} H_{u}^{'}=\sum_{i\in |{\text{Φ}}_{u}|}A_{i,u}{\text{Φ}}_{i,u}+H_{u} \end{array}$$

where, $H_{u}^{"}$ represents the feature vector at position u in the output feature map $H^{\prime }\in {\text{R}}^{C\times W\times H}$; $A_{i,u}$ is the scalar weight at channel i and position u in the attention map A.

The collected contextual information is added to the local features H to enhance the local features and expand pixel-level representation capabilities. As a result, the model gains a broader contextual view and selectively aggregates contextual information based on the spatial attention map. These feature representations mutually reinforce each other, enhancing the robustness of the network. Finally, regarding implementation details, the Criss-Cross Attention module is designed to function as a dimension-preserving component. Given an input feature map $X\in {\text{R}}^{\text{C}\times H\times W}$, the module produces an output feature map $Y\in {\text{R}}^{\text{C}\times H\times W}$. Notably, both the channel depth ( C) and the spatial resolution ( H×W) remain strictly unchanged during this process, ensuring that the module focuses solely on enhancing pixel-level representation through global context aggregation without altering any tensor dimensions.

#### 2.4.4 ContextGuidedBlock_Down module

In semantic segmentation tasks, particularly when processing high-resolution aerial images, the encoder inevitably encounters the issue of spatial detail loss during the layer-wise downsampling process. Traditional dimension-reduction operations, such as max pooling, while effectively expanding the receptive field and reducing computational load, inherently involve a lossy compression of spatial information. For the task at hand, critical fine-grained features—such as the intricate, serrated boundary contours of *Fagus* crowns and small potential saplings within forested areas—are highly prone to being overlooked or blurred during this process. This loss of high-frequency detail directly compromises the model’s ability to achieve fine-grained segmentation, resulting in coarse boundaries and merged instances.

**Figure 9:**
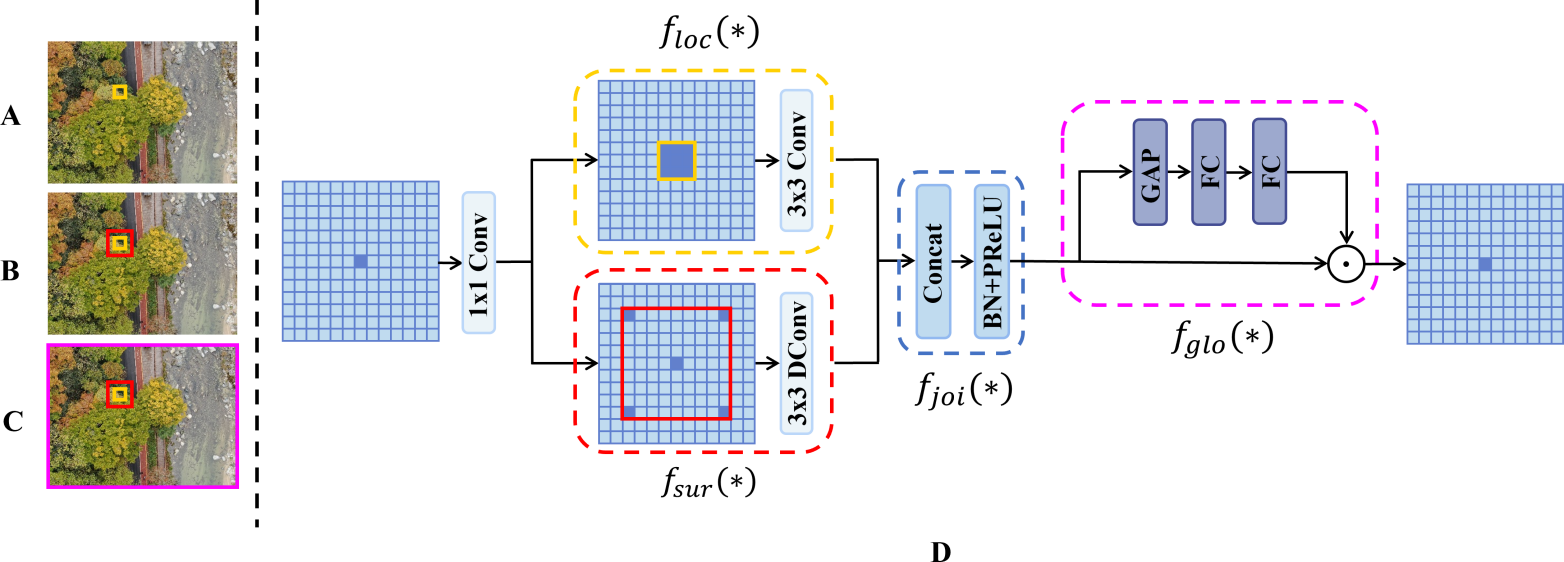
ContextGuidedBlock_Down module. **(A)** It is difficult to categorize the yellow region when we only pay attention to the yellow region itself. **(B)** It is easier to recognize the yellow region with the help of its surrounding context (red region). **(C)** Intuitively, we can categorize the yellow region with a higher degree of confidence when we further consider the global contextual information (purple region). **(D)** The structure of ContextGuidedBlock_Down block, which consists of local feature extractor $f_{loc}(*)$,surrounding context extractor $f_{sur}(*)$, joint feature extractor $f_{glo}(*)$.

Although employing large convolutional kernels can enhance contextual modeling capabilities to some extent, compensating for partial information loss, it significantly increases the model’s parameter count and computational overhead, which contradicts the goal of creating efficient models. Therefore, the challenge becomes how to optimize the downsampling process to actively preserve fine-grained spatial information while maintaining a lightweight design. To address this, this study introduces the Context-Guided Block (CGB) (see **Figure 9**). This module is specifically architected to support fine-grained segmentation by retaining local structural details (via local feature extractors) while simultaneously capturing broader context, thereby ensuring that jagged leaf edges and individual crown boundaries remain distinct even after downsampling.

The ContextGuidedBlock_Down block is designed to fully leverage the inherent property of semantic segmentation. The most inherent property of semantic segmentation is that contextual information is crucial for pixel-level recognition, as inspired by the human visual system. As shown in **Figure 9A**, suppose the human visual system attempts to identify pixels located within the yellow region. If we focus solely on this area, classification becomes difficult due to its small size and insufficient information. As illustrated in **Figure 9B**, we define the red region as the surrounding context of the yellow region. When both the yellow region and its surrounding context are considered, classifying the yellow region becomes significantly easier. This is because the red region covers a larger area than the yellow region and contains more useful information. Therefore, the surrounding context is beneficial for semantic segmentation. In **Figure 9C**, if the human visual system further captures the global context of the entire scene (the purple area) and combines it with the yellow region and its surrounding context (the red area), it can achieve higher confidence in classification. This is because the global context provides a holistic representation of the scene, which positively influences the identification of every pixel within it. Thus, both the surrounding and global context contribute to improving segmentation accuracy.

Based on the above analysis, the ContextGuidedBlock_Down block consists of a local feature extractor $f_{loc}(*)$, a surrounding context extractor $f_{sur}(*)$, a joint feature extractor $f_{joi}(*)$, and a global context extractor $f_{glo}(*)$. The module comprises two main steps: the feature learning step and the feature improvement step.

In the feature learning step, the local feature extractor $f_{loc}(*)$ and the surrounding context extractor $f_{sur}(*)$ are used to learn local features and the corresponding surrounding context features, respectively. Specifically, $f_{loc}(*)$ is instantiated as a 3×3 standard convolution layer that learns local features from the eight-neighborhood, corresponding to the yellow region in **Figure 9A**. Meanwhile, $f_{sur}(*)$ is instantiated as a 3×3 dilated convolution layer; thanks to its larger receptive field, it effectively captures surrounding context, corresponding to the red region in **Figure 9B**. Subsequently, the joint feature extractor $f_{joi}(*)$ fuses the local and surrounding context features from the outputs of $f_{loc}(*)$ and $f_{sur}(*)$. To reduce computational complexity, the ContextGuidedBlock_Down block implements $f_{joi}(*)$ as a concatenation layer followed by batch normalization (BN) and parametric ReLU (PReLU) operations.

During the feature improving step, the global context extractor $f_{glo}(*)$ extracts global context to enhance the joint features. Inspired by SENet (Hu et al., 2018), the global context is treated as a weighted vector and applied at the channel level to fine-tune the joint features, emphasizing useful components while suppressing irrelevant ones. In practice, this module implements $f_{glo}(*)$ as a global average pooling layer followed by a multilayer perceptron (MLP). The global average pooling layer efficiently aggregates the global context of the scene image, corresponding to the purple region in **Figure 9C**. Finally, the ContextGuidedBlock_Down block employs a channel-wise scaling layer to use the extracted global context to reweight the joint features. Notably, $f_{glo}(*)$ is adaptive to the input image, as the extracted global context is generated from that image. Thus, the feature improving step demonstrates advantages similar to attention mechanisms. Furthermore, the feature learning step focuses on learning joint features in the spatial dimension, while the feature improving step concentrates on optimizing the joint features in the channel dimension, meaning that the two steps are orthogonal. Channel-level feature improvement from the global context extractor can emphasize useful channels and suppress uninformative ones, thereby helping to improve segmentation accuracy. Finally, regarding implementation details, the ContextGuidedBlock_Down module is explicitly designed to function as a spatial downsampling component, replacing standard pooling layers. Given an input feature map $X\in {\text{R}}^{C_{in}\times H\times W}$, the module produces an output feature map $Y\in {\text{R}}^{C_{out}\times \frac{H}{2}\times \frac{W}{2}}$. Notably, this operation performs a strict reduction in spatial resolution by a factor of 2, while simultaneously expanding the channel dimension (typically $C_{out}>C_{in}$) to encode richer semantic information, ensuring that fine-grained details are preserved despite the geometric compression.

### 2.5 Model training

#### 2.5.1 Experimental detail

The experimental training for this study was conducted on a system equipped with an NVIDIA RTX 3080 Ti GPU. The primary software environment used in the experiments includes PyTorch 1.8.1, Python 3.8, CUDA 11.1, torch 1.2.0, and torchvision 0.4.0.

Other hyperparameter configurations are shown in **Table 2**.

**Table 2:** Hyperparameter settings for the experiment.

Hyperparameter	Details
Epochs	100
Image size	512 × 512
Batch size	8
Workers	4
Optimizer	Adam
Seed	11
lr0	1e-7
lr1	1e-9
lrf	Cosine (ratio=0.01)

#### 2.5.2 Loss function

In this study, Cross-Entropy Loss was used during model training. This loss function is widely applied in image segmentation tasks, particularly for pixel-level classification problems. Cross-Entropy Loss measures the difference between the model’s predictions and the actual labels, effectively guiding the model to learn accurate pixel classification. Its mathematical expression is given in Equation 14:

(14) $$\begin{array}{c} \text{CE}\left(p,q\right)=-\sum_{i=1}^{C}y_{i}\log\left({\hat{y}}_{i}\right) \end{array}$$

where *C* represents the total number of categories, i is the category index, $y_{i}$ is the one-hot encoded vector of the true label, and ${\hat{y}}_{i}$ is the predicted probability distribution output by the model. The loss function calculates the discrepancy between the predicted probability for each pixel category and the actual label, thereby driving the model to progressively optimize its classification ability during training.

### 2.6 Evaluation metrics

This paper employs mIoU, mPA, mPrecision, Recall, and Model Size as primary evaluation metrics. Here, “m” stands for “mean,” indicating the average across all categories, used to measure the model’s overall performance in multi-class segmentation tasks. The definitions and calculation formulas for each metric are shown in **Table 3**.

**Table 3:** Parameter definitions for evaluation metrics.

Expected results	Predicted results	
	Positive	Negative
True	TP	FN
False	FP	TN

TP (True Positive): Positive samples that are correctly predicted as positive by the model.

TN (True Negative): Negative samples that are correctly predicted as negative by the model.

FP (False Positive): Negative samples that are incorrectly predicted as positive by the model.

FN (False Negative): Positive samples that are incorrectly predicted as negative by the model.

IoU: It is the most commonly used evaluation metric in semantic segmentation tasks, used to measure the overlap between the predicted result and the ground truth area, in order to assess the accuracy of the segmentation results, as defined in Equation 15.

(15) $$\begin{array}{c} IOU=\frac{\left|target\cap prediction\right|}{\left|target\cup prediction\right|} \end{array}$$

PA: The ratio of correctly classified pixels to the total number of pixels in the model, used to measure the overall pixel classification accuracy, as defined in Equation 16.

(16) $$\begin{array}{c} PA=\frac{TP+TN}{TP+TN+FP+FN} \end{array}$$

Precision: The proportion of pixels correctly identified as belonging to the positive class among all pixels that the model classifies as belonging to the positive class, as defined in Equation 17.

(17) $$\begin{array}{c} Precision=\frac{TP}{TP+FP} \end{array}$$

Recall: The proportion of pixels correctly identified as belonging to the positive class among all pixels that actually belong to the positive class, as defined in Equation 18.

(18) $$\begin{array}{c} Recall=\frac{TP}{TP+FN} \end{array}$$

Model Size: Represents the storage space occupied by the model’s parameter count, used to measure the model’s complexity and deployment overhead.

## 3 Results

### 3.1 Comparison experiment of data augmentation strategies

**Figure 10:**
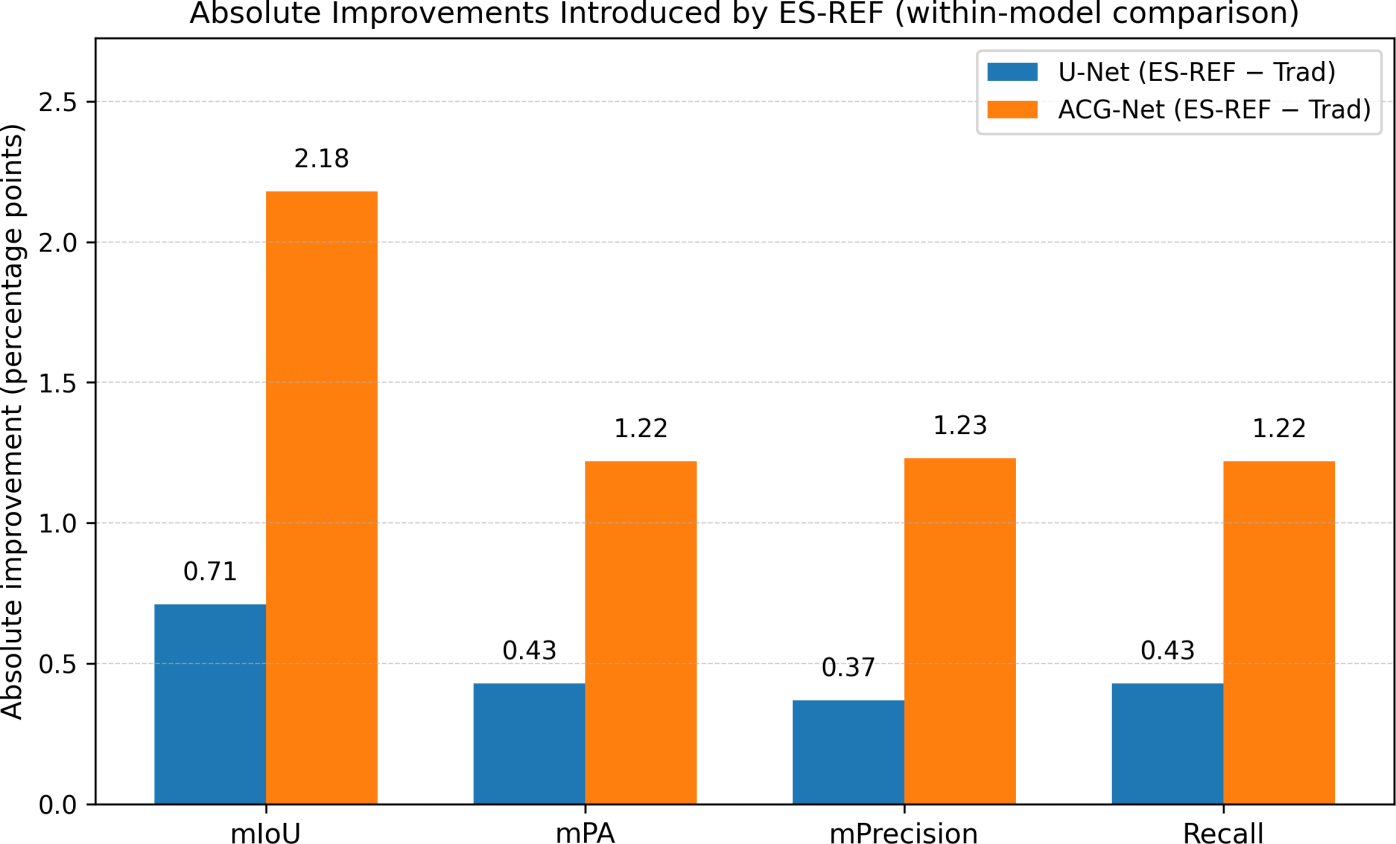
Absolute improvements introduced by ES-REF over traditional augmentations (ES-REF − Trad) for U-Net and ACG-Net, measured by mIoU, mPA, mPrecision, and Recall under identical training settings.

To isolate the contribution of the Environment Simulation-based Robust Data Augmentation Framework (ES-REF), we performed a controlled comparison against standard augmentation techniques while keeping all training protocols identical. The resulting performance differences are summarized in **Figure 10**.

**Figure 10** quantifies the absolute performance gains achieved by replacing traditional augmentations with ES-REF. For the baseline U-Net, ES-REF yields improvements across all metrics, with mIoU increasing by 0.71 percentage points, mPA and Recall by 0.43 percentage points each, and mPrecision by 0.37 percentage points. For ACG-Net, mIoU increases by 2.18 percentage points, mPA and Recall by 1.22 percentage points each, and mPrecision by 1.23 percentage points.

**Table 4** presents the complete set of numerical results. Under traditional augmentation, U-Net achieves an mIoU of 84.53% and a model size of 94.93 MB. With ES-REF, its mIoU rises to 85.24% and model size to 94.97 MB. For ACG-Net, traditional augmentation yields an mIoU of 87.37% and a model size of 100.71 MB. When trained with ES-REF, ACG-Net reaches an mIoU of 89.55% while maintaining the same model size.

**Table 4:** Comparison of data augmentation strategies.

Model	mIoU	mPA	mPrecision	Recall	Model size
Unet - Traditional Data Augmentation	84.53	91.60	91.67	91.60	94.93
Unet - Environment Simulation-based Robust Augmentation	85.24	92.03	92.04	92.03	94.97
ACG-Net - Traditional Data Augmentation	87.37	93.26	93.26	93.26	100.71
ACG-Net - Environment Simulation-based Robust Augmentation	89.55	94.48	94.49	94.48	100.71

### 3.2 Comparison experiment of the convolutional part

To validate the impact of different convolution structures on model performance, this study compares several advanced convolution operators, including TiedBlockConv (Wang and Yu, 2021), CondConv (Yang et al., 2019), RefConv (Cai et al., 2025b), ODConv (Li et al., 2022), DOConv (Cao et al., 2022), and the SPConv used in this study. All models were trained and evaluated under the same conditions, and the experimental results are shown in **Table 5**.

**Table 5:** Comparison of the convolutional part.

Model	mIoU	mPA	mPrecision	Recall	Model size
Unet(baseline)	85.24	92.03	92.04	92.03	94.97
Unet-TiedBlockConv	86.24	92.60	92.65	92.60	94.95
Unet-CondConv	86.78	92.92	92.92	92.92	207.25
Unet- RefConv	85.60	92.24	92.25	92.24	151.07
Unet- ODConv	83.29	90.90	90.89	90.90	264.59
Unet-DOConv	86.25	92.62	92.62	92.62	97.27
Unet-SPConv	87.71	93.44	93.49	93.44	73.33

The baseline U-Net achieves an mIoU of 85.24% with a model size of 94.97 MB. Among the convolution variants, mIoU values range from 83.29% to 87.71%, and model sizes range from 73.33 MB to 264.59 MB. SPConv achieves an mIoU of 87.71% with a model size of 73.33 MB. TiedBlockConv attains an mIoU of 86.24% with a model size of 94.95 MB. CondConv reaches an mIoU of 86.78% with a model size of 207.25 MB. RefConv yields an mIoU of 85.60% with a model size of 151.07 MB. DOConv achieves an mIoU of 86.25% with a model size of 97.27 MB. ODConv produces an mIoU of 83.29% with a model size of 264.59 MB.

### 3.3 Comparison experiment of the attention mechanism

To evaluate the impact of different attention mechanisms on model performance, this study integrated several mainstream attention modules, including DoubleAttention (Chen et al., 2018b), ECAAttention (Wang et al., 2020), SEAttention, CBAM (Woo et al., 2018), ACmix (Pan et al., 2021), CoordAtt (Hou et al., 2021), and the Criss-CrossAttention module selected in this study. All models were trained and evaluated under identical conditions, and the experimental results are shown in **Table 6**.

**Table 6:** Comparison of the attention mechanism part.

Model	mIoU	mPA	mPrecision	Recall	Model size
Unet(baseline)	85.24	92.03	92.04	92.03	94.97
Unet-DoubleAttention	86.14	92.56	92.55	92.56	97.96
Unet-ECAAttention	86.50	92.76	92.77	92.76	94.94
Unet-SEAttention	86.49	92.75	92.76	92.75	95.19
Unet-CBAM	86.18	92.58	92.57	92.58	95.10
Unet-ACmix	86.44	92.72	92.74	92.72	98.14
Unet-CoordAtt	86.64	92.83	92.87	92.83	95.07
Unet-Criss-CrossAttention	87.17	93.15	93.15	93.15	96.22

Among the attention variants, mIoU values range from 86.14% to 87.17%, and model sizes range from 94.94 MB to 98.14 MB. U-Net with Criss-Cross Attention achieves an mIoU of 87.17% with a model size of 96.22 MB. U-Net with DoubleAttention attains an mIoU of 86.14% with a model size of 97.96 MB. U-Net with ECAAttention achieves an mIoU of 86.50% with a model size of 94.94 MB. U-Net with SEAttention attains an mIoU of 86.49% with a model size of 95.19 MB. U-Net with CBAM yields an mIoU of 86.18% with a model size of 95.10 MB. U-Net with ACmix reaches an mIoU of 86.44% with a model size of 98.14 MB. U-Net with CoordAtt achieves an mIoU of 86.64% with a model size of 95.07 MB.

### 3.4 Comparison experiment of the downsampling part

To validate the impact of different downsampling strategies on model performance, this study compares several advanced downsampling modules, including Adown (Wang et al., 2024), HWD (Xu et al., 2023), RHDWT (Yuan et al., 2025), SPPDown (He et al., 2015), SPPFDown, and the ContextGuidedBlock_Down (CGB_Down) module chosen for this study. All models were trained and evaluated under identical conditions, and the experimental results are shown in **Table 7**.

**Table 7:** Comparison of the downsampling part.

Model	mIoU	mPA	mPrecision	Recall	Model size
Unet(baseline)	85.24	92.03	92.04	92.03	94.97
Unet- ADown	86.91	92.99	93.03	92.99	100.83
Unet- HWD	87.11	93.10	93.13	93.10	104.33
Unet- RHDWT	84.68	91.70	91.71	91.70	199.80
Unet- SPPDown	87.25	93.18	93.22	93.18	138.05
Unet- SPPFDown	87.01	93.04	93.07	93.04	138.05
Unet- ContextGuidedBlock_Down	88.10	93.68	93.67	93.68	121.10

Among the downsampling variants, mIoU values range from 84.68% to 88.10%, and model sizes range from 100.83 MB to 199.80 MB. U-Net with CGB_Down achieves an mIoU of 88.10% with a model size of 121.10 MB. U-Net with SPPDown attains an mIoU of 87.25% with a model size of 138.05 MB. U-Net with HWD achieves an mIoU of 87.11% with a model size of 104.33 MB. U-Net with SPPFDown reaches an mIoU of 87.01% with a model size of 138.05 MB. U-Net with ADown achieves an mIoU of 86.91% with a model size of 100.83 MB. U-Net with RHDWT yields an mIoU of 84.68% with a model size of 199.80 MB.

### 3.5 Ablation study

**Figure 11:**
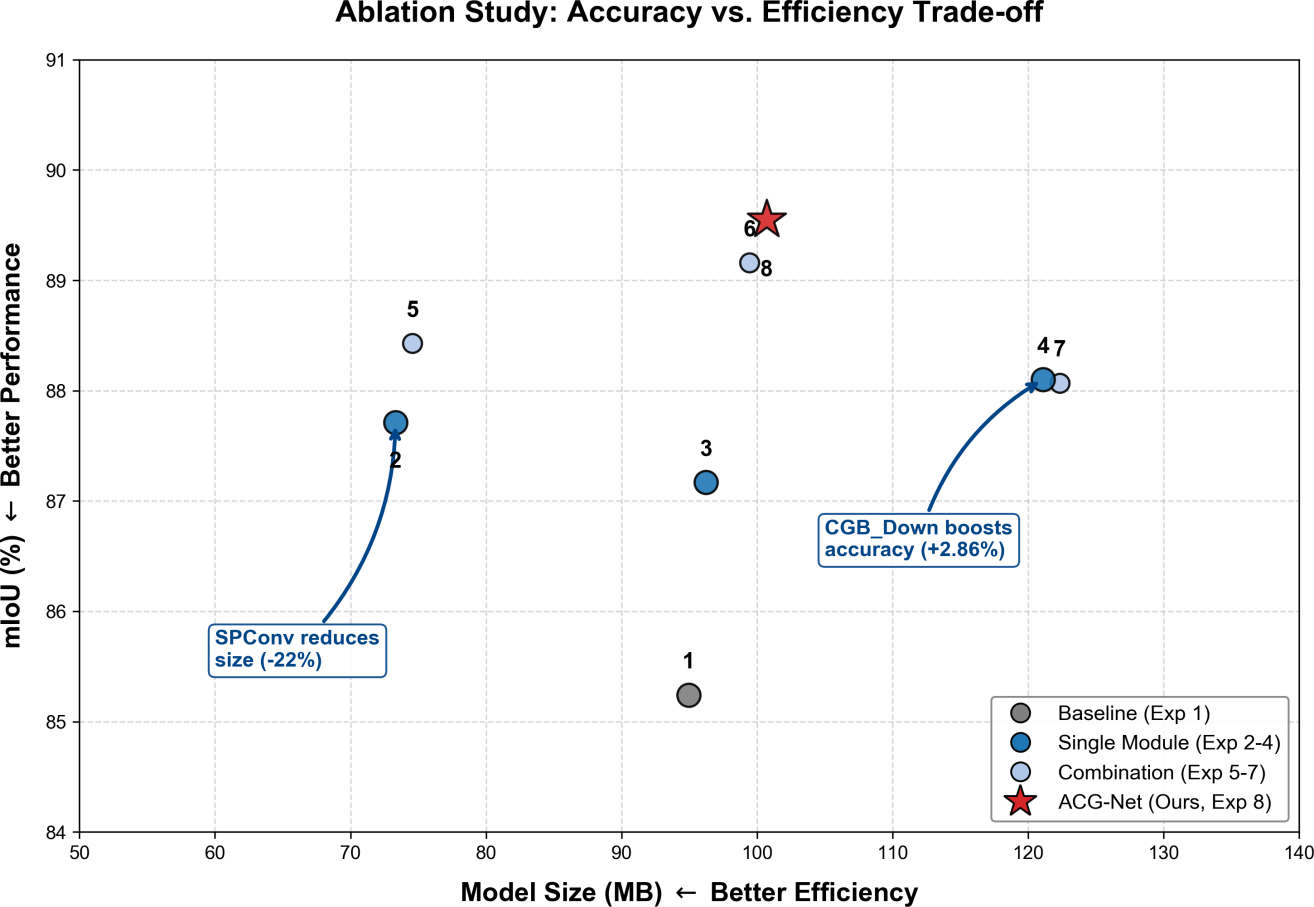
Ablation study visualization: Trade-off between accuracy and model size. Scatter plot of mIoU (%) versus model size (MB) for the ablation experiments. Numbered markers correspond to the experiment IDs listed in **Table 8** (e.g., 1 = baseline; 2–4 = single-module experiments; 5–7 = module combinations; 8 = full ACG-Net). Marker colors and shapes denote experiment groups as indicated in the legend. Higher positions correspond to higher mIoU (better performance), whereas leftward positions correspond to smaller model size (better efficiency).

To systematically validate the effectiveness of the proposed improvement modules, we use the U-Net as the baseline model and design a series of ablation experiments. This section aims to independently assess the contributions of the three main modules—SPConv, Criss-CrossAttention, and ContextGuidedBlock_Down (CGB_Down)—to model performance, and further explore their synergistic effects when used together. All experiments were conducted in a standardized environment, with detailed results shown in **Table 8**, where “√” indicates that the module was integrated, and “×” indicates that it was not. the trade-off between segmentation performance and model efficiency is visualized in **Figure 11**.

**Table 8:** Ablation study comparison.

Numbers	SPConv	Criss-CrossAttention	CGB_Down	mIoU	mPA	mPrecision	Recall	Model size
1	×	×	×	85.24	92.03	92.04	92.03	94.97
2	✓	×	×	87.71	93.44	93.49	93.44	73.33
3	×	✓	×	87.17	93.15	93.15	93.15	96.22
4	×	×	✓	88.10	93.68	93.67	93.68	121.10
5	✓	✓	×	88.43	93.86	93.86	93.86	74.58
6	✓	×	✓	89.16	94.25	94.31	94.25	99.45
7	×	✓	✓	88.07	93.66	93.65	93.66	122.35
8	✓	✓	✓	89.55	94.48	94.49	94.48	100.71

The baseline U-Net (Experiment 1) achieves an mIoU of 85.24% with a model size of 94.97 MB. With SPConv alone (Experiment 2), mIoU reaches 87.71% and model size is 73.33 MB. With Criss-Cross Attention alone (Experiment 3), mIoU reaches 87.17% and model size is 96.22 MB. With CGB_Down alone (Experiment 4), mIoU reaches 88.10% and model size is 121.10 MB.

With SPConv and Criss-Cross Attention combined (Experiment 5), mIoU reaches 88.43% with a model size of 74.58 MB. With SPConv and CGB_Down combined (Experiment 6), mIoU reaches 89.16% with a model size of 99.45 MB. With Criss-Cross Attention and CGB_Down combined (Experiment 7), mIoU reaches 88.07% with a model size of 122.35 MB.

With all three modules combined—ACG-Net (Experiment 8)—mIoU reaches 89.55% with a model size of 100.71 MB. The corresponding mPA, precision, and recall are 94.48%, 94.49%, and 94.48%, respectively.

### 3.6 Comparison experiment with mainstream models

To comprehensively evaluate the overall performance of the proposed ACG-Net for the fine-grained segmentation task of *Fagus*, we conducted a systematic comparison with various mainstream semantic segmentation networks, including U-Net, DeepLabv3+ (Chen et al., 2018a), PSPNet (Zhao et al., 2017), HRNet (Wang et al., 2021), and SegFormer (Xie et al., 2021). The experimental results are shown in **Table 9**.

**Table 9:** Comparison with mainstream models.

Model	mIoU	mPA	mPrecision	Recall	Model size
Unet	85.24	92.03	92.04	92.03	94.97
deeplabv3-plus	85.12	91.92	92.20	91.92	209.71
PSPnet	85.28	92.03	92.12	92.03	178.49
Hrnet	87.30	93.21	93.23	93.21	113.57
Segformer	86.85	92.96	92.97	92.96	104.46
ACG-Net (ours)	89.55	94.48	94.49	94.48	100.71

Among the evaluated models, mIoU values range from 85.12% to 89.55%, and model sizes range from 94.97 MB to 209.71 MB. ACG-Net achieves an mIoU of 89.55% with a model size of 100.71 MB. U-Net achieves an mIoU of 85.24% with a model size of 94.97 MB. DeepLabv3+ achieves an mIoU of 85.12% with a model size of 209.71 MB. PSPNet achieves an mIoU of 85.28% with a model size of 178.49 MB. HRNet achieves an mIoU of 87.30% with a model size of 113.57 MB. SegFormer achieves an mIoU of 86.85% with a model size of 104.46 MB.

**Figure 12:**
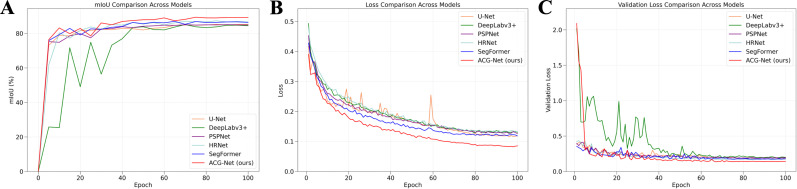
Performance comparison of different models. **(A)** mIoU comparison of different models; **(B)** Loss comparison of different models; **(C)** Validation loss comparison of different models.

**Figure 13:**
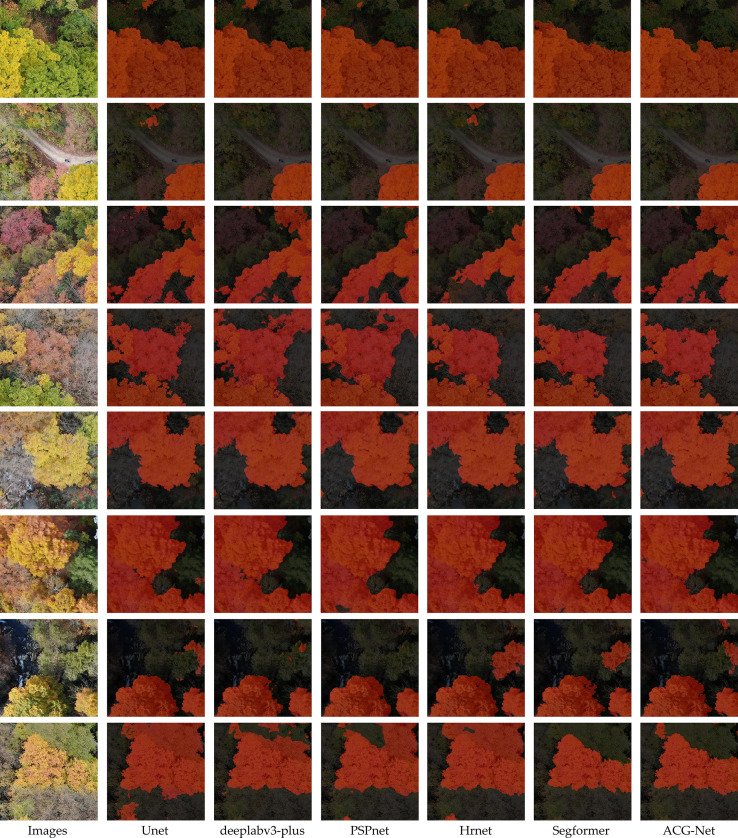
Comparison of prediction results from different models.

**Figure 12** plots the training trajectories of all compared models, illustrating the evolution of mIoU, training loss, and validation loss throughout the optimization process. **Figure 13** presents qualitative comparisons of segmentation outputs on representative aerial imagery.

## 4 Discussion

The primary motivation of this study was to address the specific limitations of standard architectures, such as U-Net, in extracting fine-grained features from complex forest scenes. Our experimental results indicate that ACG-Net effectively addresses these challenges by establishing a synergistic balance between efficiency, global context modeling, and local detail preservation. This improvement appears to result from the collaborative function of three key modules. To mitigate feature redundancy in high-resolution imagery, the SPConv module replaces standard convolutions to reduce parameter redundancy, allowing the model to learn discriminative features without the heavy parameter burden seen in redundant architectures. To distinguish *Fagus* from spectrally similar co-occurring species and complex understory backgrounds, the Criss-Cross Attention module captures long-range dependencies by aggregating contextual information along both horizontal and vertical spatial directions. This capacity to model long-range spatial dependencies across the entire feature map is particularly useful for separating *Fagus* crowns from spectrally similar backgrounds over large spatial extents, where local context alone may be insufficient and local convolutions struggle to capture large-scale structure. Furthermore, the ContextGuidedBlock_Down (CGB_Down) module specifically targets the “serrated edge” loss caused by pooling. By leveraging contextual guidance to adaptively preserve high-frequency details during downsampling, CGB_Down helps retain the intricate boundary morphology of *Fagus* even in deep layers, improving boundary consistency. Compared with multi-scale pooling strategies (e.g., SPPDown, HWD) that primarily emphasize context aggregation or frequency decomposition, CGB_Down is designed to preserve fine-grained boundary structures during spatial resolution reduction, addressing the tendency of pooling or strided convolutions to discard the high-frequency information that defines intricate canopy edges.

The ablation study in **Table 8** quantifies the individual and combined contributions of the three modules. Relative to the baseline U-Net (85.24% mIoU, 94.97 MB), SPConv alone (Experiment 2) increases mIoU to 87.71% while reducing model size to 73.33 MB, indicating improved parameter efficiency. CGB_Down alone (Experiment 4) yields the largest single-module accuracy gain (mIoU 88.10%) with a larger model (121.10 MB). Criss-Cross Attention alone (Experiment 3) achieves a moderate gain (87.17% mIoU) with a minimal size increase (96.22 MB). When SPConv and CGB_Down are combined (Experiment 6), mIoU reaches 89.16% with a model size of 99.45 MB—a size between those of the individual modules—suggesting that SPConv’s lightweight design can partially offset the parameter cost of CGB_Down while retaining most of its accuracy benefit. Adding Criss-Cross Attention to this pair (Experiment 8) further raises mIoU to 89.55% with a slight increase in model size (100.71 MB), indicating that global context modeling complements local detail preservation and efficient feature extraction. These results suggest that the three modules target distinct but complementary aspects of the segmentation task—computational efficiency, boundary detail preservation, and global context aggregation—and that their integration yields a balanced improvement in both accuracy and model compactness.

Beyond architectural optimizations, the proposed ES-REF framework addresses the domain gap caused by environmental interferences. In practical forest monitoring, visibility reduction from fog and texture distortion from motion blur are frequent issues that standard augmentations do not fully model. Our experiments show that ES-REF improves the segmentation accuracy of ACG-Net, suggesting the benefit of incorporating targeted environment simulation in training. By simulating physically plausible degradations during training, ES-REF expands the data distribution in a directed manner, encouraging the model to learn features more invariant to contrast attenuation and brightness distortion. This degradation-aware strategy is closer to the actual imaging process in forestry scenarios than generic geometric transformations, thereby improving reliability under non-ideal field conditions. As shown in **Table 4**, applying ES-REF increases ACG-Net’s mIoU from 87.37% to 89.55% without altering model size or inference cost—an improvement that highlights the effectiveness of this training-only augmentation approach.

In comparison with mainstream models, ACG-Net presents a favorable balance between accuracy and model complexity. While DeepLabv3+ and PSPNet are strong benchmarks, their larger model sizes may limit suitability for resource-constrained forestry applications. In our experiments, ACG-Net attains higher mIoU with substantially smaller parameter size than these heavyweight models, which indicates that SPConv contributes to parameter reduction while maintaining accuracy. ACG-Net also compares favorably to architectures such as HRNet and the Transformer-based SegFormer. Although SegFormer employs global self-attention, ACG-Net achieves higher accuracy with a slightly smaller model size, implying that for fine-grained canopy segmentation generic attention mechanisms may miss some local morphological details. Combining Criss-Cross Attention for global context with CGB_Down for local detail preservation thus provides a focused solution for identifying the irregular, serrated boundaries of *Fagus* canopies.

Despite these promising results, several limitations merit discussion. First, the approach depends on the representativeness of the annotated training data; the current study focused on a specific geographic region during the autumn leaf-coloration period, which may aid identification but limits generalization to other seasons (e.g., summer) or different forest types. Second, although ES-REF models key degradations, real-world disturbances can be more diverse and involve complex couplings not fully captured by current simulations. Third, while ACG-Net shows a favorable accuracy-size trade-off for offline processing, deployment on resource-constrained edge devices (e.g., drone-embedded chips) for real-time inference remains challenging and may require additional compression techniques such as quantization or pruning.

Future research will pursue three main directions to address these challenges and broaden application value. First, we will explore semi-supervised or weakly supervised learning to reduce reliance on dense pixel-level annotations and enhance scalability for large-scale forest surveys. Second, to overcome seasonal limitations, we plan to integrate multi-source remote sensing data, such as LiDAR point clouds and infrared imagery; this multimodal approach is expected to improve discrimination in season-neutral scenarios. Finally, beyond species identification, we aim to extend the framework to fine-grained physiological health monitoring. By analyzing intra-canopy spectral variations (e.g., stress-induced discoloration) and integrating infrared-derived vegetation indices, we intend to develop automated chlorophyll monitoring and early disease detection, thereby enhancing “smart forestry” applications and supporting sustainable resource management.

## 5 Conclusion

To address the multifaceted challenges of feature redundancy, insufficient context, and environmental interference in fine-grained *Fagus* canopy segmentation, this study establishes a holistic framework comprising the novel ACG-Net and the ES-REF augmentation strategy. By synergistically integrating SPConv, Criss-Cross Attention, and Context-Guided Downsampling, ACG-Net effectively optimizes computational efficiency while simultaneously capturing global dependencies and preserving fine-grained spatial details. Complementing this architectural optimization, the ES-REF framework actively bridges the domain gap by simulating physical degradations, thereby ensuring robustness against real-world disturbances. Consequently, our method achieved a state-of-the-art mIoU of 89.55%, significantly outperforming standard baselines and providing a reliable methodological foundation for automated forest resource monitoring and sustainable ecosystem management.

## Data Availability

The raw data supporting the conclusions of this article will be made available by the authors, without undue reservation.
